# A conserved arginine within the αC-helix of Erk1/2 is a latch of autoactivation and of oncogenic capabilities

**DOI:** 10.1016/j.jbc.2023.105072

**Published:** 2023-07-18

**Authors:** Nadine Soudah, Alexey Baskin, Karin Smorodinsky-Atias, Jonah Beenstock, Yifat Ganon, Ruchama Hayouka, Mohammed Aboraya, Oded Livnah, Ronit Ilouz, David Engelberg

**Affiliations:** 1 Department of Biological Chemistry, The Institute of Life Science, The Hebrew University of Jerusalem, Jerusalem, Israel; 2 School of Neurobiology, Biochemistry and Biophysics, Tel Aviv University, Tel Aviv-Yafo, Israel; 3 The Robert H. Smith Institute of Plant Sciences and Genetics in Agriculture, The Hebrew University of Jerusalem, Rehovot, Israel; 4 The Azrieli Faculty of Medicine, Bar Ilan University, Safed, Israel; 5 The Wolfson Centre for Applied Structural Biology, Jerusalem, Israel; 6 Singapore-HUJ Alliance for Research and Enterprise, Mechanisms of Liver Inflammatory Diseases Program, National University of Singapore, Singapore; 7 Department of Microbiology and Immunology, Yong Loo Lin School of Medicine, National University of Singapore, Singapore

**Keywords:** ERK, MAP kinase, autophosphorylation, eukaryotic protein kinases, active variants, activation loop

## Abstract

Eukaryotic protein kinases (EPKs) adopt an active conformation following phosphorylation of a particular activation loop residue. Most EPKs spontaneously autophosphorylate this residue. While structure–function relationships of the active conformation are essentially understood, those of the “prone-to-autophosphorylate” conformation are unclear. Here, we propose that a site within the αC-helix of EPKs, occupied by Arg in the mitogen-activated protein kinase (MAPK) Erk1/2 (Arg84/65), impacts spontaneous autophosphorylation. MAPKs lack spontaneous autoactivation, but we found that converting Arg84/65 of Erk1/2 to various residues enables spontaneous autophosphorylation. Furthermore, Erk1 molecules mutated in Arg84 are oncogenic. Arg84/65 thus obstructs the adoption of the “prone-to-autophosphorylate” conformation. All MAPKs harbor an Arg that is equivalent to Arg84/65 of Erks, whereas Arg is rarely found at the equivalent position in other EPKs. We observed that Arg84/65 of Erk1/2 interacts with the DFG motif, suggesting that autophosphorylation may be inhibited by the Arg84/65–DFG interactions. Erk1/2s mutated in Arg84/65 autophosphorylate not only the TEY motif, known as critical for catalysis, but also on Thr207/188. Our MS/MS analysis revealed that a large proportion of the Erk2^R65H^ population is phosphorylated on Thr188 or on Tyr185 + Thr188, and a small fraction is phosphorylated on the TEY motif. No molecules phosphorylated on Thr183 + Thr188 were detected. Thus, phosphorylation of Thr183 and Thr188 is mutually exclusive suggesting that not only TEY-phosphorylated molecules are active but perhaps also those phosphorylated on Tyr185 + Thr188. The effect of mutating Arg84/65 may mimic a physiological scenario in which allosteric effectors cause Erk1/2 activation by autophosphorylation.

Eukaryotic protein kinases (EPKs) are involved in controlling all aspects of life and therefore have been thoroughly studied. Consequently, important aspects of the mechanism of catalysis and structure–function relationships of these enzymes are known in detail (1, 2, 3, 4). Yet, critical issues of regulation, mainly by self-activation, are not understood well (5). This study shows that a particular position within EPKs, which was hitherto less appreciated, is a central element in mitogen-activated protein kinase (MAPK) autoactivation and perhaps in regulation of all EPKs. Our attention to this site was drawn by the fact that in the protein kinase Erk1, mutating the residue at this position renders the kinase oncogenic (6).

Although EPKs could be divided to several subfamilies with unique catalytic and structural properties (1, 7), they all share a similar topological fold comprising two lobes. A smaller N-lobe is composed of a core of four beta-strands (β1–β4) and a single α-helix (known as the αC-helix), whereas a larger C-lobe is predominantly α-helical. The N-lobe harbors the ATP-binding site, and the C-lobe includes the substrate-binding site and the catalytic loop, which contains the catalysis essential motif, HRD (4, 8, 9, 10).

The catalytic activity of EPK is tightly regulated. A large number of specific modes of regulation has been acquired by the different EPKs, but almost all of them share a basic activation mechanism, namely, phosphorylation of a residue; in most EPKs, a threonine (Thr197 in cyclic AMP–dependent PKA [cPKA]), located within a highly conserved segment known as the activation loop (11, 12). The activation loop contains essential sequences, such as the DFG motif, which binds Mg/ATP (9, 11). Phosphorylation of the activation loop imposes dramatic conformational changes, which result in converting the kinase fold from an “inactive” to an “active conformation.” Two structural entities, known as the regulatory spine and catalytic spine (C-spine), are assembled in the “active conformation” (8, 13, 14, 15). The regulatory spine consists of four residues. Two come from the C-lobe, including the Phe of the DFG and the His of the HRD motifs. The other two are aliphatic residues located in the N-lobe’s αC-helix and β4 strand (8). The C-spine is comprised of eight hydrophobic residues, two from the N-lobe and six from the C-lobe. The assembly of the C-spine is completed upon ATP binding rendering the kinase capable of catalysis (13, 14).

A hallmark of the active conformation following activation loop phosphorylation is provided by a particular Glu residue of the αC-helix, which forms a salt bridge with a Lys residue of the β3. Mutating this Glu in cPKA results in total elimination of catalytic activity (14, 16). This αC-helix’s Glu is highly conserved amongst EPKs and is absent from only 33 typical kinases of 497 human kinases that we analyzed. The αC-helix further contributes to stabilization of the active conformation by its movement and formation of bonds with other regions of the kinase (3, 17).

Another consequence of activation loop phosphorylation is formation of H-bond interaction between the phosphorylated Thr and a His residue in the αC-helix (His87 in cPKA) (18). His87 is not conserved amongst EPKs, and the role of its interaction with phos-Thr97 is not clear. This interaction was proposed to assist in stabilizing spine assembly (14), but mutating His87 to Ala results in an approximately threefold increase in the catalytic rate of cPKA (14, 19), suggesting that the interaction of phos-Thr197 with His87 is not a critical element of the active conformation and may have in fact a negative effect on catalysis. In Erk1 and Erk2, the position occupied by His87 in cPKA is occupied by an Arg. This Arg (84 and 65 in Erk1 and Erk2, respectively) is the focus of this study. We propose that the residue at this position is important for adoption of yet another conformation of EPKs, the “prone-to-autophosphorylate” conformation (5).

Phosphorylation of the activation loop’s Thr is commonly catalyzed by another EPK, but most EPKs are capable, to various degrees, of spontaneously autophosphorylating (5, 11, 20, 21). The ability to autophosphorylate implies, by definition, that EPKs acquire, in addition to the nonactive and active conformations, a conformation that supports autophosphorylation, termed a “prone-to-autophosphorylate” conformation (5). This conformation is required for a single-round reaction with a single intramolecular substrate, because once autophosphorylation occurs, no further substrate exists and the kinase adopts an active conformation. The “prone-to-autophosphorylate” conformation is therefore short-lived, and its structure is not fully understood (5).

Erk1 and Erk2 form a subgroup within the MAPK family. MAPKs, which also include the c-Jun N-terminal kinase (JNK), p38, and ERK5/BMK groups (22, 23), are structurally similar to all other EPKs but possess certain features that distinguish them. Most of them are incapable of spontaneous autophosphorylation, namely do not acquire the “prone-to-autophosphorylate” conformation. Phosphorylation of their activation loop, and concomitant activation, is dependent on specific and dedicated kinases known as MAPK kinases (MAPKKs; also termed MAP2K, MEKs, or MKKs) (24). Notably, MAPKs do possess an inherent capacity of autophosphorylation, but, as mentioned, it is not spontaneous but rather inhibited or occluded. It can be derepressed through mutations (5, 25, 26, 27, 28, 29, 30) or, in the specific instances of p38α, p38β, or p38γ, by physiologically relevant mechanisms (31, 32, 33). The activation of MAPK requires not a single but a dual phosphorylation of the activation loop on a TXY motif (T^202/183^EY^204/185^ in Erk1/Erk2) to become fully active. The structural features that prevent Erk1/2 (and all MAPKs) of spontaneously adopting the “prone-to-autophosphorylate” conformation are not clarified.

Erks are activated *via* the receptor tyrosine kinases–Ras–Raf–MEK cascade, which is constitutively active in a large number of cancers because of activating (oncogenic) mutations (34). Erks themselves are rarely found mutated in these diseases (35). Mutations reported to render Erks active, primarily gain-of-function mutants, were found to possess no intrinsic catalytic capabilities ([*e.g.*, *sevenmaker* (36), mutations identified in cancer patients [E320K in ERK2] [mutation ID in ERK2, COSM461148] (35, 37)). They seem to bestow a gain-of-function property because of their reduced affinity to phosphatases (38, 39). A handful of *bona fide* intrinsically active mutants were identified in the laboratory and include Erk2^S151D^ (30), Erk2^I84A^ (26), and Erk1^R84S^/Erk2^R65S^ (6, 25). Of these, only Erk1^R84S^/Erk2^R65S^ were shown to be spontaneously active *in vitro*, in mammalian cells in culture, and in transgenic flies and mice (6, 25, 35, 40, 41). Erk1^R84S^ was further shown to oncogenically transform NIH3T3 cells (6). Importantly, another mutation in Arg84 of ERK1, R84H, was identified in two cancer patients (mutation ID: COSM4875436) and also in a screen for mutations that render cells that are transformed by oncogenic Raf resistant to Raf and MEK inhibitors (42). The effects of the R84H mutation on the biochemical or biological properties of Erk1 were not reported, and it is not known whether Erk1^R84H^ plays a causative role in the tumors in which it is found.

The mechanism that renders Erk1^R84S^ and Erk2^R65S^ independent of upstream activation is the acquirement of spontaneous autophosphorylation/autoactivation capability (6, 25), suggesting that the mutation allows “prone-to-autophosphorylate” conformers. Erk1^R84S^ and Erk2^R65S^ autophosphorylate not only their TEY motif but also another phosphoacceptor within the activation segment, Thr207 in Erk1 and Thr188 in Erk2 (6). Thr207/188 phosphorylation has been observed in a mouse model and in human patients of cardiac hypertrophy (43). The role of Thr207/188 phosphorylation, if any, in Erks’ regulation, and specifically in establishment of the “prone-to-autophosphorylate” conformation of Erk1^R84S^ and Erk2^R65S^ is not known. Here, we report that a rigorous MS/MS analysis did not identify molecules that are phosphorylated on Thr183 + Thr188 in the population of Erk2^R65S^, whereas many molecules within this population are phosphorylated on Tyr185 + Thr188, or (seem less abundant) on Thr183 + Tyr185.

The acquirement of autophosphorylation capability of Erk1^R84S^ and Erk2^R65S^ and the oncogenic potential of Erk1^R84S^ were the impetus for investigating the role of Arg84/65 in depth. Here, we provide evidence that this Arg is a “locker” of Erks' intrinsic activity and of its oncogenicity. Replacement of this Arg by various other residues, including His, found in tumors, renders the resulting Erk1 molecules oncogenic. It is also reported that all MAPKs throughout evolution, with no exception, possess an Arg at this position. Despite its conservation, in the Hog1/p38 and JNK groups, this Arg plays a somewhat different role than in Erks. Other protein kinases harbor varying residues at this position but rarely an Arg. Structural analysis revealed that Arg84/65 of Erk interacts with the DFG motif, providing a potential explanation for its ability to occlude autophosphorylation.

Thus, the position within the αC-helix occupied by Arg in all MAPKs and by His in cPKA seems to play a central role in determining the kinase’s autoactivation properties. In the case of Erk1, it also determines its oncogenic capability. The residue at this position is specifically involved in autoactivation and is dispensable for adopting the active conformation and for catalysis *per se*.

## Results and discussion

### Mutating Arg84/Arg65 of Erk1/2 to His or to other residues renders the kinases intrinsically active and oncogenic

Two possible general structure–function explanations may underlie the self-activation ability of Erk1^R84S^ and Erk2^R65S^. One notion suggests that the Arg to Ser mutation enforces a precise and specific “prone-to-autophosphorylate” conformation. Another possible mechanism is that the Arg84/65 residue blocks autophosphorylation, and therefore, its replacement with other residues, not only Ser, would render the kinase capable of autophosphorylation. To distinguish between these possibilities, Arg84 in Erk1 and Arg65 in Erk2 were replaced with Ser, His, Ala, Tyr, Thr, Pro, or Lys, and the resulting proteins were tested in three assays: (1) *in vitro* kinase assay as recombinant proteins; (2) phosphorylation status of the TEY motif when expressed in human embryonic kidney 293T (HEK293T) cells; and (3) capability to oncogenically transform NIH3T3 cells.

**Figure 1:**
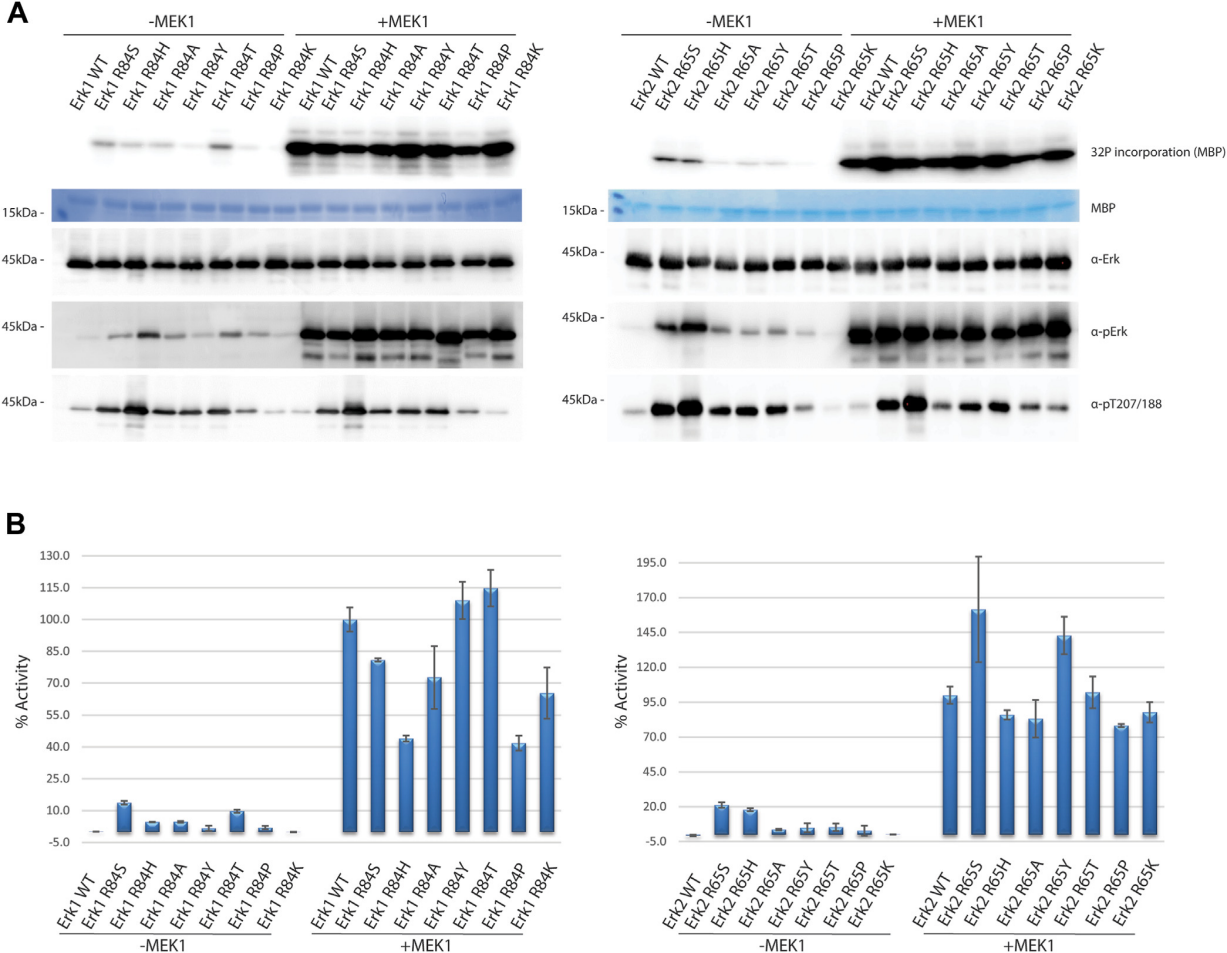
**Mutating Arg84/Arg65 of Erk1/2 to various other residues renders the resulting mutants intrinsically active *in vitro*.** The indicated recombinant purified Erk1/2 mutants were tested in a kinase assay with or without pretreatment with active MEK1. *A*, fixed volume from each reaction was subjected to SDS-PAGE and stained with *Coomassie brilliant blue* to verify loading equal amounts of MBP (*second row*) and was then exposed to a phosphorimager screen to visualize ^32^P incorporation (*first row*). Samples from each reaction were also subjected to Western blot analysis and probed with antibodies against total Erk1/2 (*third row*), phosphorylated(TEY)-Erk1/2 (*fourth row*), and phosphorylated(Thr207/Thr188)-Erk1/2 (*fifth row*). *B*, a portion of each kinase assay reaction was quantified in a β-counter. Quantifications are shown as percentage of the activity of MEK1-activated Erk1/2^WT^, which was considered as 100% ± standard deviation (100% of Erk1^WT^ = 235,264 cpm; 100% of Erk2^WT^ = 145,430 cpm). Reactions were performed in triplicates. MBP, myelin basic protein.

*In vitro* kinase assays with purified recombinant proteins, phosphorylated or not by MEK, revealed that all mutated proteins, except Erk1^R84K^/Erk2^R65K^, manifested an intrinsic (Mek-independent) catalytic activity (Fig. 1, *A* and *B*). Activity levels were not equal among mutants. Erk1^R84S^ and Erk2^R65S^ manifested the highest intrinsic activity, reaching 14.8 ± 0.94% (mean ± SD) and 21.2 ± 1.98%, of the activity of MEK1-phosphorylated Erk1^WT^ or Erk2^WT^, respectively, as measured in a scintillation counter. Erk1^R84H^ exhibited an intrinsic catalytic activity of 4.6 ± 0.16% and Erk2^R65H^ of 17.9 ± 1.02% (Fig. 1*B*). Other mutants showed lower Mek-independent activity (Fig. 1, *A* and *B*). Intriguingly, although intrinsically active, and although efficiently phosphorylated by MEK1 (Fig. 1*A*, *fourth row*), some of the mutants manifested reduced MEK1-dependent activity (Fig. 1, *A* and *B*). These include Erk1^R84H^ whose activity was as low as 44% as compared with that of MEK-activated Erk1^WT^ (Fig. 1*B*). The low MEK1-dependent activity of Erk1^R84P^/Erk2^R65P^ may be a result of the effect of proline on the αC-helix conformation, whereas the low activity of Erk1^R84K^/Erk2^R65K^ may suggest that Lys, which is relatively similar to the native Arg residue in this position, is a more powerful latch of activity than Arg. The lower MEK1-dependent activity of Erk1^R84H^/Erk2^R65H^ and Erk1^R84S^ is more difficult to explain. It is speculated that these mutants adopt a stable “prone-to-autophosphorylate” conformation and do not convert into the optimal “active conformation” fold even following phosphorylation by MEK1. It is not clear to us in this regard why Erk2^R65S^ manifests 161% activity when phosphorylated by MEK1.

Intrinsic activity of the mutants was found to be correlated, in general, with the level of spontaneous phosphorylation on the TEY motif (Fig. 1*A*, *fourth row*; for Erk2^R65S^ and Erk2^R65H^, see also verification of TEY phosphorylation by MS/MS, Table 1). Namely, while Erk1/2^WT^ and Erk1/2^R84/65/K^ proteins are weakly dually phosphorylated on the TEY motif when purified from *Escherichia coli* cells, Erk1/2^R84/65/S^ and Erk1/2^R84/65/H^ are dually phosphorylated stronger than all other mutants. Intrinsic activity is also correlated with the level of phosphorylation on Thr207/188 (Fig. 1*A*, *fifth row*; Table 1). Notably, incubation of the proteins with active MEK1, which increased activity toward myelin basic protein significantly, did not affect the levels of Thr207/188 phosphorylation (Fig. 1*A*, *fifth row*). These observations clearly point at autophosphorylation as the sole source of Thr207/188 phosphorylation and at strong correlation between Thr207/188 phosphorylation and autophosphorylation of the TEY motif.

**Table 1:** Intensities of the indicated peptides in each of the Erk2 proteins shown, as measured in tandem mass spectrometry

Peptide modified sequence	ERK2^WT^	ERK2^R65S^	ERK2^R65H^
	Intensity^a^		
VADPDHDHTGFLTEYVATR	7.4 × 10^9^	3.5 × 10^8^	1.7 × 10^8^
VADPDHDHTGFL**pT**EYVATR	0.00	2.2 × 10^6^	1.7 × 10^6^
VADPDHDHTGFLTE**pY**VATR	2.1 × 10^9^	1.0 × 10^10^	9.1 × 10^9^
VADPDHDHTGFLTEYVA**pT**R	3.4 × 10^6^	1.0 × 10^7^	1.5 × 10^7^
VADPDHDHTGFL**pT**E**pY**VATR	0.00	2.2 × 10^7^	7.3 × 10^7^
VADPDHDHTGFL**pT**EYVA**pT**R	0.00	0.00	0.00
VADPDHDHTGFLTE**pY**VA**pT**R	2.4 × 10^6^	6.0 × 10^7^	2.9 × 10^8^
VADPDHDHTGFL**pT**E**pY**VA**pT**R	0.00	0.00	2.4 × 10^6^

The bold values in the table represent the residues that were found to be phosphorylated in the peptide.

a Values represent peptide intensities normalized to total Erk2 abundance, as calculated based on measured nonmodified Erk2 peptides GQVFDVGPR, FDMELDDLPK, and ICDFGLAR.

**Figure 2:**
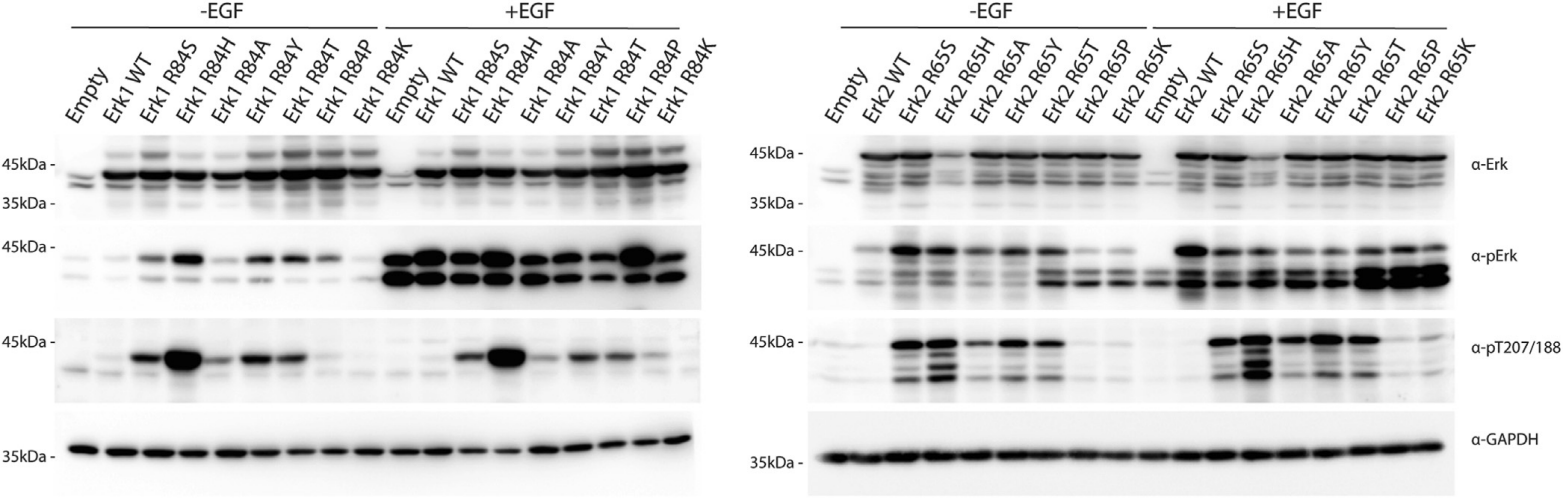
**Erk1/2 molecules mutated in Arg84/Arg65 are spontaneously phosphorylated in HEK293T cells.** Expression vectors carrying the indicated Erk1/2 mutants were introduced into HEK293T cells. About 48 h post-transfection, cells were serum starved for 16 h, and EGF was added to the indicated plates (50 ng/ml for 10 min). Protein lysates were then prepared and subjected to Western blot analysis and probed with antibodies against total Erk1/2 (*first row*), phosphorylated(TEY)-Erk1/2 (*second row*), phosphorylated(Thr207/Thr188)-Erk1/2 (*third row*), and GAPDH (*fourth row*). Note that the externally expressed Erk1 molecules (tagged with polyhistidine that adds just 0.8 kDa to the molecular weight) migrate very close to, almost overlap with, the endogenous Erk1 (*left panel*). Externally expressed Erk2 molecules, tagged with HA run slower than the endogenous Erk1 and Erk2, as the tag adds 3.2 kDa to the molecular weight (*right panel*). EGF, epidermal growth factor; HA, hemagglutinin; HEK293T, human embryonic kidney 293T cell line.

**Supplemental Fig. S1:**
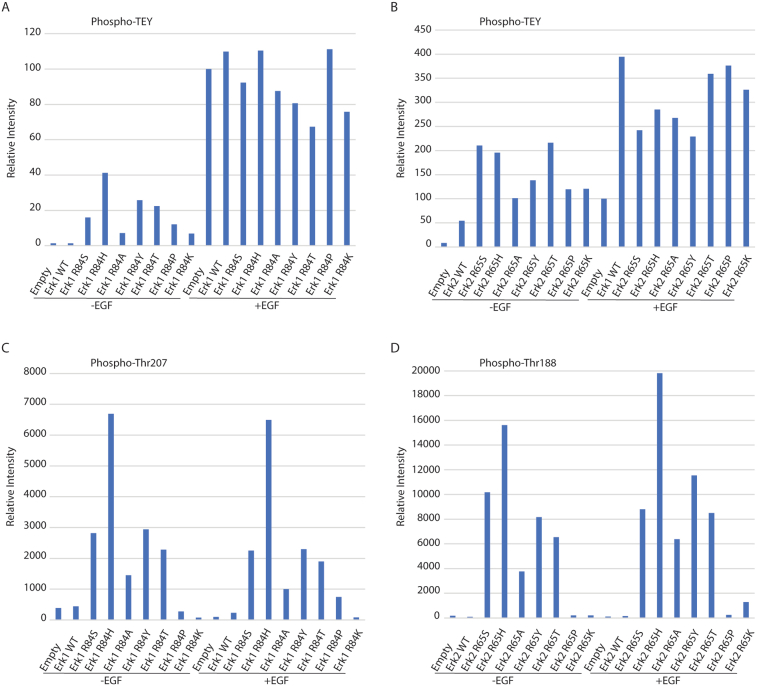
**Relative quantification of the western blot data shown in**Figure 2**, as measured by ImageJ.** A & B) Quantification of phospho-TEY levels. C & D) Quantification of phospho-Thr207/188. Measurements were normalized to phosphorylation levels of measured in cells harboring an empty plasmid.

**Supplemental Fig. S2:**
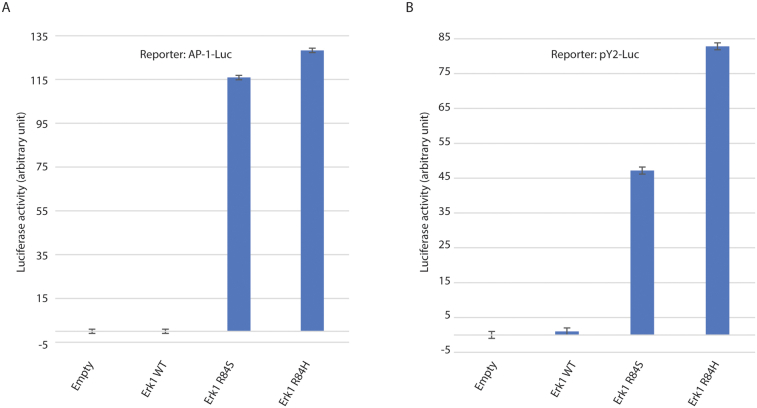
**Expression of Erk1**^**R84S**^**and Erk1**^**R84H**^**induces strong AP-1-mediated transcription in HEK293T cells.** Plasmids expressing Erk1^R84S^ and Erk1^R84H^ were co-introduced into HEK293T cells with either 6xAP-1-luciferase (A) or pY2-luciferase (B) reporter genes. Cells were collected 24 h later and assayed for luciferase activity, which was normalized to activity of co-expressed renilla luciferase.

To test whether the intrinsic activity is also manifested in mammalian cells, the Erk1/2 mutants were transiently expressed in HEK293T cells. While Erk1/2^WT^ proteins were phosphorylated at low levels in serum-starved cells that were not exposed to any stimulus, most mutants (except Erk1^R84P^/Erk2^R65P^ and Erk1^R84K^/Erk2^R65K^) were spontaneously phosphorylated on the TEY motif (Fig. 2, *second row*) as well as on Thr207/188 (Fig. 2, *third row*). We further tested the most active variants of Erk1, Erk1^R84S^, and Erk1^R84H^, for their functionality, by testing their effect on AP-1 transcriptional activity. Both proteins strongly activated AP-1-mediated reporter genes (Fig. S2). Curiously, the less active Erk2 mutants, Erk2^R65T^, Erk2^R65P^, and Erk2^R65K^, caused an increase in TEY phosphorylation of the endogenous Erk1/2 (Fig. 2, *second row*, *right blot*). The mechanism of this effect is currently unclear. In accordance with the observation with the purified proteins, in which Thr207/188 phosphorylation was not affected by exposure to active recombinant MEK1, exposure of HEK293T to epidermal growth factor (EGF) caused dramatic elevation in TEY phosphorylation but did not affect Thr207/188 phosphorylation whatsoever (Fig. 2, *third row*). Phosphorylation levels of TEY and Thr207/188 were quantified (Fig. S1).

**Figure 3:**
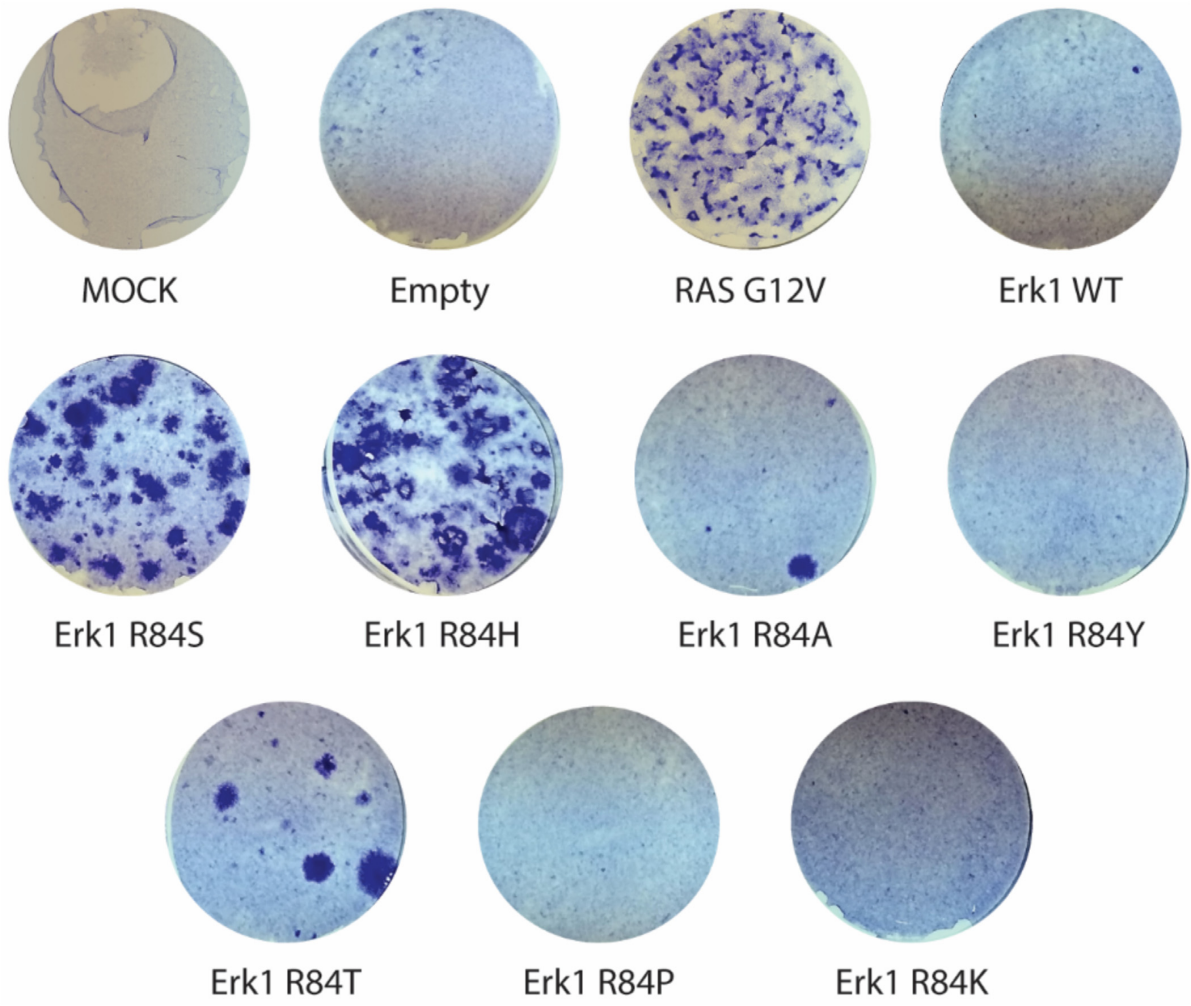
**Some of the Erk1 molecules mutated at Arg84 oncogenically transform NIH3T3 cells.** Expression vectors carrying the indicated Erk1/2 mutants were introduced into NIH3T3 cells. Expression vectors carrying no gene (empty vector) or carrying Erk1^WT^ were used as negative controls, whereas a vector expressing the oncogenic H-RAS^G12V^ was used as a positive control for cell transformation. Cells expressing the indicated vectors were selected by the addition of G418 and fixed and stained with crystal violet 4 weeks after transfection. Statistical analysis revealed that the differences in oncogenic efficiency between Erk1^R84S^ and Erk1^R84H^ mutants were not statistically significant (*p* value of 0.36). Similarly, no significant differences were observed when comparing Erk1^R84A^ with Erk1^R84Y^, Erk1^R84T^, Erk1^R84P^, and Erk1^R84K^ mutants (average *p* value of 0.16). However, the remaining samples exhibited statistically significant differences, with an average *p* value of 0.023.

It was next tested whether the unregulated catalytic activity of the new mutants may impose an oncogenic effect, similar to that reported for Erk1^R84S^ (6). Erk1^R84H^ was found able to transform NIH3T3 cells to a similar degree as Erk1^R84S^, with 25 and 20 foci (≥2 mm radius) per microgram of transfected DNA, respectively (Fig. 3). Given that the R84H mutation is identified in adenocarcinoma and endometrioid carcinoma (mutation ID: COSM4875436), this observation suggests that Erk1^R84H^ could be the disease-causing oncoprotein. In agreement with the *in vitro* kinase assays, Erk1^R84T^ and Erk1^R84A^ were less efficient oncogenes than Erk1^R84S^ and gave rise to six and two foci per microgram of DNA, respectively. Erk1^R84K^, Erk1^R84P^, and Erk1^R84Y^ did not give rise to appearance of foci (Fig. 3). In contrast to the Erk1 mutants, none of the Erk2 mutants was able to transform these cells as not even a single focus appeared to grow over the contact-inhibited cell layer following their expression.

### Replacing Arg68 of the yeast Erk/Mpk1 to Ser, His, Ala, Tyr, Thr, and Glu renders it independent of MEKs

**Figure 4:**
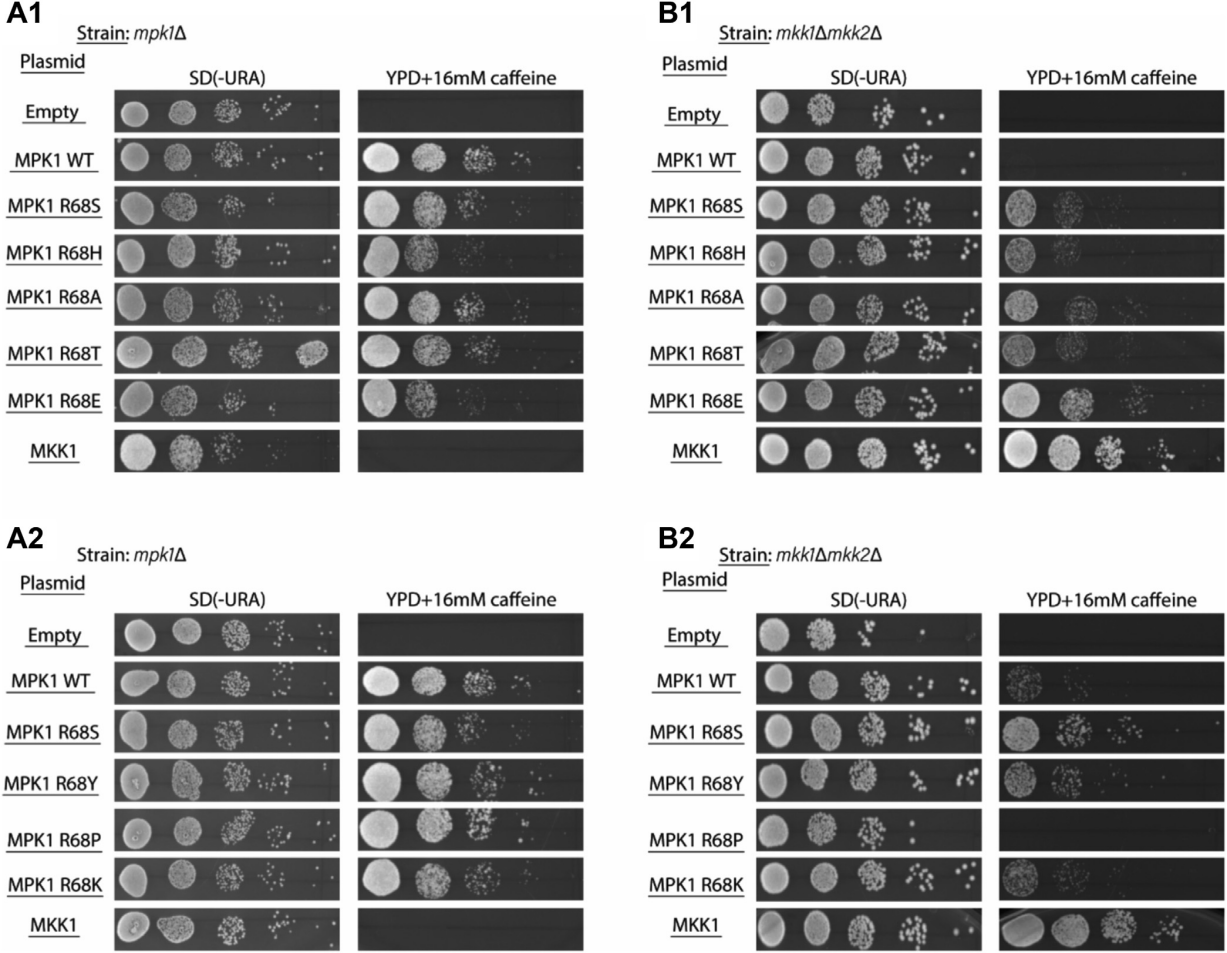
**Various mutants of the yeast MAPK Erk/Mpk1, mutated at Arg68 (but not Mpk1**^**R68K**^**and Mpk1**^**R68P**^**), are intrinsically active.** Plasmids carrying the indicated mutants were introduced to *mpk1Δ* yeast cells (*A*) or *mkk1Δmkk2Δ* cells (*B*). Transformants were grown to logarithmic phase on SD (−URA) medium and then plated in serial dilutions (1:10) on either SD (−URA) plates or on plates containing YPD supplemented with 16 mM caffeine, as indicated. In panels *A1* and *B1* mutants were expressed from YEP352, and in panels *A2* and *B2*, mutants were expressed from AES426 expression vector. MAPK, mitogen-activated protein kinase; YPD, yeast extract–peptone–dextrose.

Erk1^R84S^ and Erk2^R65S^ were created on the basis of a mutation, R68S, which was identified in a genetic screen in yeast that searched for mutants of the MAPK Erk/Mpk1 that rescue *mkk1Δmkk2Δ* cells from sensitivity to caffeine (25). Mpk1 is essential for the synthesis and maintenance of the yeast cell wall, so that yeast strains knocked out for MPK1 or for the genes encoding its MAPKKs, called MKK1 and MKK2 (*i.e.*, *mpk1Δ* and *mkk1Δmkk2Δ* strains, respectively), do not grow in the presence of compounds that damage the cell wall (*e.g.*, caffeine) (44, 45). To test whether in accordance with the mammalian Erk1 and Erk2, mutating Arg68 of Mpk1 to various residues (and not just to Ser) would render the kinase MKK1/2 independent, Arg68 was replaced with His, Ala, Tyr, Thr, Glu, Pro, or Lys. All Mpk1 mutants were able to rescue *mpk1Δ* cells showing that none of the changes of R68 affected the activity (MKK1/2 dependent) of the enzyme (Fig. 4, *A1* and *A2*). When expressed in *mkk1Δmkk2Δ* cells, most mutants were able to rescue the cells from caffeine stress (Fig. 4, *B1* and *B2*), suggesting that similar to the observation in the mammalian Erk1/2, mutating the particular Arg residue within the αC-helix to various residues renders the yeast Mpk1 too intrinsically active. Also similar to Erk1/2, replacing Arg68 with Pro or Lys did not render Mpk1 intrinsically active (Fig. 4, *B1* and *B2*).

The fact that all Mpk1 mutants, including Mpk1^R68P^ and Mpk1^R68K^, rescued *mpk1Δ* cells strengthens the notion that the αC-helix Arg of the Erks is dispensable for catalysis *per se* and serves as a suppressor of intrinsic autoactivation.

### Arg65 of Erk2 is associated with the DFG motif

The results described previously support the notion that the position occupied by Arg84/Arg65/Arg68 in Erk1/2/Mpk1 is a hotspot for mutations that evoke spontaneous catalytic and biological activities of the entire ERK family. It seems that an Arg was selected by evolution for this position in Erk proteins to suppress autocatalysis. Lysine, which shares chemical properties with Arg, is also an efficient suppressor of autophosphorylation.

**Figure 5:**
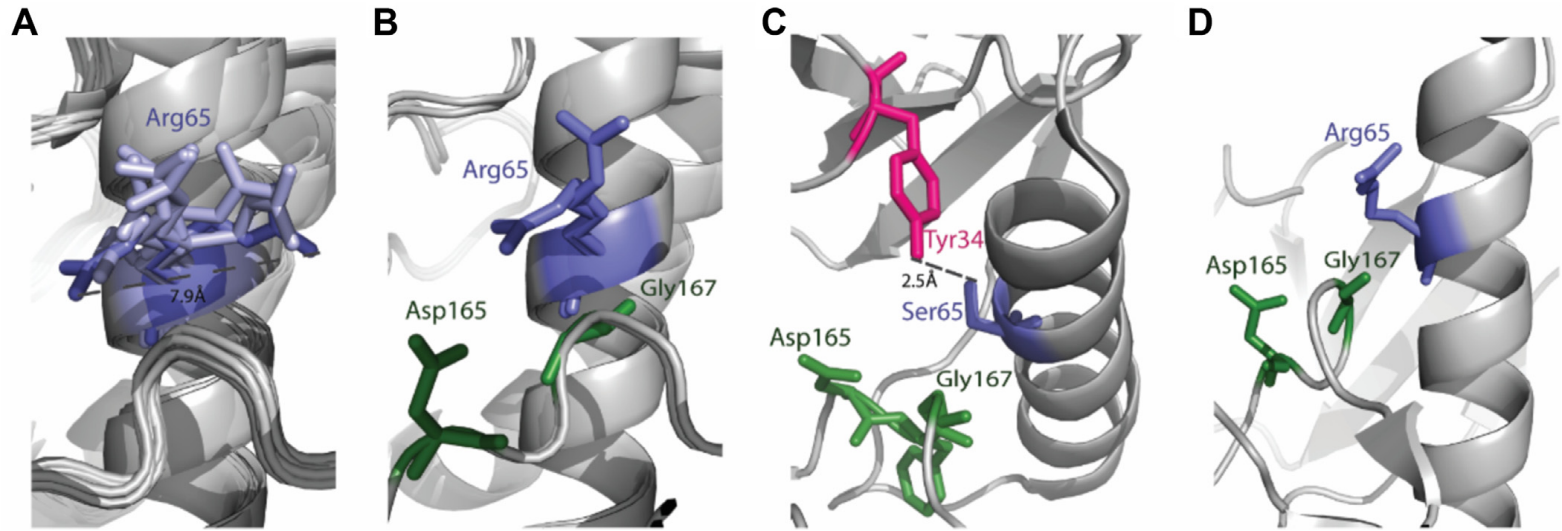
**Arg65 of Erk2 may block spontaneous autophosphorylation activity *via* its interaction with the DFG motif.***A*, superposition of Arg65 from nine crystal structures (Protein Data Bank codes: 1ERK, 3ERK, 4ERK, 4S31, 4GT3,5UMO, 6RFP, 6FLE, and 4XRL) of ERK2 that were crystallized in the P21 space group and have similar cell parameters. The two extreme conformations are colored in *dark purple*. *B*, Arg65 conformation comparison between two Erk2^WT^ structures (Protein Data Bank codes: 5UMO and 4S31). In both conformations, Arg65 interacts with residues of the DFG motif, either Asp165 or Gly167. *C*, in Erk2^R65S^, Ser65 does not interact with the DFG. *D*, in Erk2^I84A^, also an intrinsically active variant (26), Arg65 does not interact with the DFG motif.

What could be the structural basis for the function of the αC-helix Arg as an obstructer of autophosphorylation? Arg65 in Erk2 is very flexible and able to accommodate many orientations (35). When comparing nine crystal structures of Erk2, which were crystallized in the P21 space group and have similar cell parameters, the measured distance between Nη of the two extreme orientations is 7.9 Å (Protein Data Bank [PDB] codes: 1ERK, 3ERK, 4ERK, 4S31, 4GT3, 5UMO, 6RFP, 6FLE, and 4XRL) (Fig. 5*A*). Despite its various orientations, in many of them, it interacts with the DFG motif. For example, comparing two Arg conformations in Erk2^WT^ structures (PDB codes: 5UMO and 4S31), in one of them, Arg65 interacts with Asp165 side chain and in the other with Gly167 backbone of the DFG motif (Fig. 5*B*). In addition, it interacts with Asp334, Thr61, and Glu69 through the backbone. In contrast, in the crystal structure of the active variant Erk2^R65S^ (PDB code: 4S2Z), Ser65 does not interact with the DFG. In this structure, Ser65 is accommodated in an opposite position to that of Arg65 in Erk2^WT^ structures and possibly interacts with Tyr34 from the P-loop (Fig. 5*C*) (6). In yet another intrinsically active variant of Erk2, Erk2^I84A^ (PDB code: 4S30), which is capable of autophosphorylation, Arg65 is also not associated with the DFG motif, similar to the case of Erk2^R65S^ (Fig. 5*D*). Thus, the inability of the residues that replace Arg65 in the active mutants to interact with the DFG is correlated with autophosphorylation and is a potential explanation for the spontaneous autophosphorylation activity of the active mutants. We speculate that Arg65 in Erk2 interacts with the DFG motif and interferes with its function to stabilize Mg^2+^/ATP binding and blocks autophosphorylation.

The role of αC-helix Arg84/65 in the active conformation (dually TEY phosphorylated), if any, is not clear. As mentioned, the residue at this position is not part of the hydrophobic spines. Arg65 of Erk2 interacts, indirectly, with the phospho-accepting Thr183 of the TEY motif, similar to the interaction between His87 and phosphorylated Thr197 of cPKA (46). Specifically, Arg65 interacts with a water molecule (Wat544 numbering in PDB code: 2ERK) that in turn interacts with phosphorylated Thr183. In the structure of phosphorylated Erk2 bound to AMP-PNP (PDB code: 6OPG), Arg65 interacts with two water molecules, Wat504 and Wat512. On one side, the water molecules interact with Arg65, and on the other side, Wat504 interacts with the phosphorylated Thr183, and Wat512 interacts with Gly167 of the DFG, thereby allowing for an indirect interaction between Arg65 to phospho-Thr183 and phospho-Gly167. Crystal structure of dually phosphorylated Erk1 is not available.

### In Erk1/2 molecules that are mutated in Arg84/65, autophosphorylation of Thr183/202 or Thr207/188 is mutual exclusive

**Figure 6:**
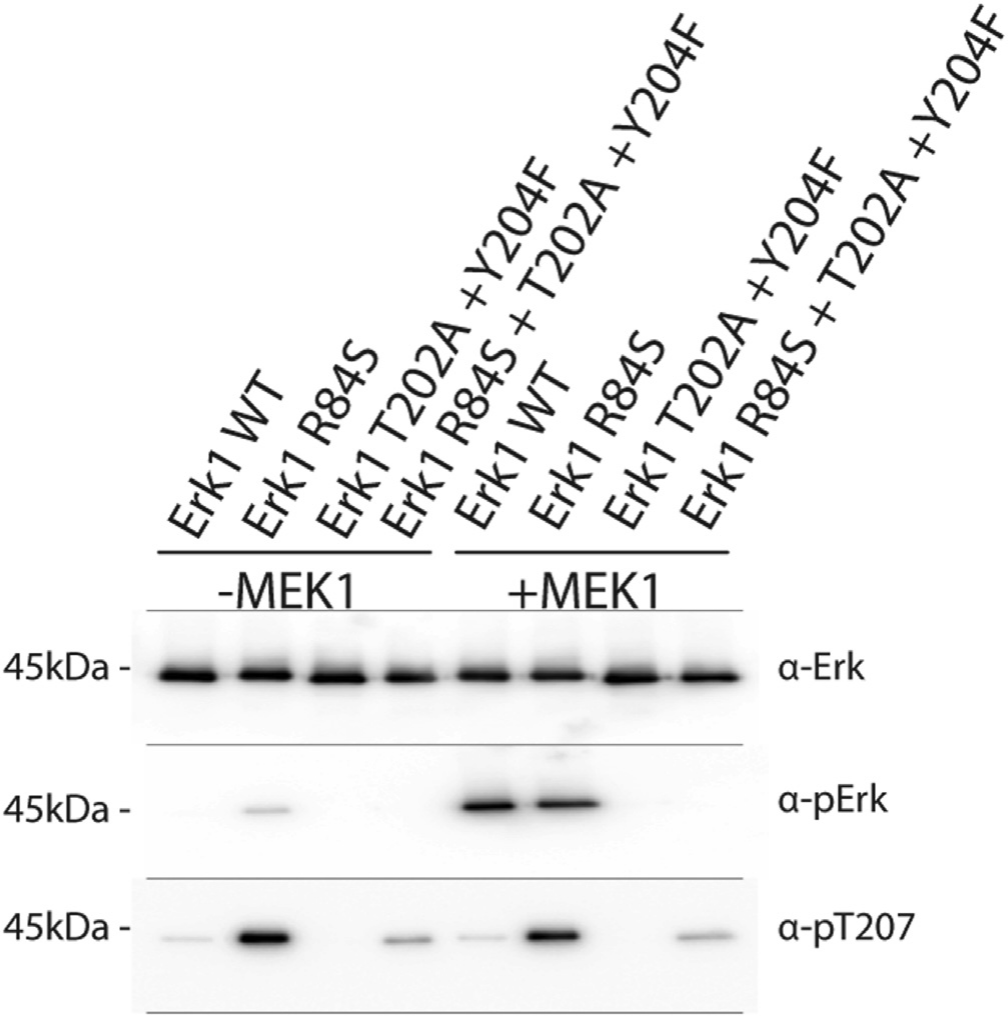
**Thr207/188 phosphorylation is independent of TEY motif phosphorylation.** The indicated recombinant purified Erk1 mutants were tested in a cold kinase assay with or without active MEK1. Fixed volume from each reaction was subjected to Western blot analysis and probed with antibodies against total Erk1/2 (*first row*), phosphorylated(TEY)-Erk1/2 (*second row*), and phosphorylated(Thr207/Thr188)-Erk1/2 (*third row*).

For Thr202/183 and Tyr204/185 of Erk1/2 to become phosphorylated, their residue’s hydroxyl must be activated and rendered nucleophilic. The base amino acid that activates all Erks’ substrates is Asp166/147, known therefore as the “catalytic Asp” (47). Crystal structures of apo Erk2 show that Asp147 interacts with Lys149 and Thr188, which is autophosphorylated in Erk2^R65S^ (Figs. 1*A* and 2) (48). The critical position of Thr188 in Erk2, and its phosphorylation in human diseases (43, 49), strongly implies that its phosphorylation plays important regulatory role, which is still unknown. The spontaneous phosphorylation of Thr207/188 in Erk1/2 molecules mutated in Arg84/65 provides an experimental platform to look into this role. The results shown previously, that Thr207/188 phosphorylation is not affected at all by MEK *in vitro*, or by EGF in living cells (Figs. 1*A* and 2), suggest that this mode of regulation is independent of the RTK–Ras–Raf–MEK pathway. Furthermore, Thr207/188 phosphorylation seems independent of TEY phosphorylation because it occurs even in molecules mutated in the TEY motif, namely, Erk1^R84S + T202A + Y204F^ (Fig. 6) and Erk2^R65S + T183A + Y185F^ (6). Thus, in the active variants, Thr207/188 phosphorylation is not mediated by the active conformation. To further inquire into the notion that Thr188 phosphorylation is independent of acquiring the active conformation *via* TEY phosphorylation, we tested whether the population of purified Erk2^WT^ proteins includes molecules that are phosphorylated only on Thr188. This was addressed *via* extensive MS/MS analysis. The identity of each phospho-peptide in the assay was unambiguously confirmed with a corresponding synthetic peptide, which contained heavy isotopes for distinguishing from the native Erk2 peptide and analyzed together by mass spectroscopy in a parallel reaction monitoring (PRM) experiment. The PRM experiment revealed that phospho-Thr188-Erk2 molecules, which are not phosphorylated on the TEY motif, do exist in the population of Erk2^WT^. There are 3-fold and 4.4-fold more such molecules in the population of Erk2^R65S^ and Erk2^R65H,^ respectively (Table 1). This confirms that phosphorylation of Thr188 is an outcome of the catalytic activity of Erk molecules that are not necessarily phosphorylated on the TEY motif. To our knowledge, no other examples of phospho-TEY-independent kinase activity for Erk have been reported.

The effect of Thr188 phosphorylation is not known yet, but regulation of Erks *via* Thr188 autophosphorylation may occur not only in the active mutants but also in the native Erk2, *in vivo*, under some physiological situations. It is plausible that there exist Erk2’s activators, perhaps allosteric, that induce a “prone-to-autophosphorylate” conformation, similar to that imposed by mutating Arg65. Subunits β and γ of the trimeric GTP-binding protein Gq are prime suspects of being such allosteric activators, as they were reported to induce Thr188 phosphorylation *in vitro* (43). This hypothetical pathway, which regulates Erk2 *via* Thr188 phosphorylation, may be overactive in patients with cardiac hypertrophy in which Thr188 is highly phosphorylated (43, 49).

**Supplemental Fig. S3:**
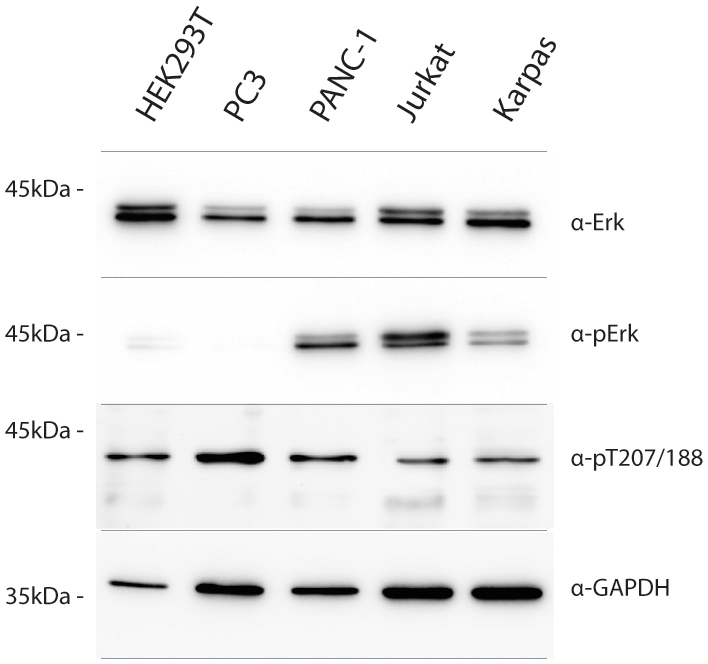
**Thr188 is phosphorylated in cancer-derived cell lines.** The indicated ancer-derived cell lines were grown to 75% confluency and collected for protein lysate preparation. 20μg of lysates were subjected to Western blot analysis using antibodies against total Erk1/2 (top row), phosphorylated(TEY)-Erk1/2 (second row), phosphorylated(Thr207/Thr188)- Erk1/2 (third row) and GAPDH (bottom row).

It is not known whether the phosphorylation of Thr188 in cardiac hypertrophy is an isolated unique case of this phosphorylation. It would be most interesting to test whether Thr188 and Thr207 are also phosphorylated in cancer patients carrying the R84H mutation in Erk1. Considering the difficulty in obtaining these extremely rare samples, we tested the cancer-derived cell lines PC3, PANC-1, Jurkat, and Karpas and observed that Erk2 is phosphorylated on Thr188, to varying levels, in all of them (Fig. S3). This phosphorylation is thus not exclusive to cardiac hypertrophy and should be further investigated in association with cancer.

As shown in Table 1, both active variants, Erk2^R65S^ and Erk2^R65H^, can autophosphorylate on Tyr185 more efficiently than Erk2^WT^ (5.1- and 4.7-fold stronger in Erk2^R65S^ and Erk2^R65H^ compared with Erk2^WT^, respectively) (Table 1). Importantly, molecules phosphorylated only on Thr183 were not detected in Erk2^WT^ population but were detected in the populations of the active variants. Also, peptides that are phosphorylated on Thr183 + Tyr185 were detected only in the active variant’s samples. Peptides phosphorylated on Tyr185 + Thr188, on the other hand, did appear at low levels in the population of Erk2^WT^ molecules and were significantly more abundant, 24.3- and 118-fold stronger, in the populations of Erk2^R65S^ Erk2^R65H^, respectively (Table 1).

Although absolute quantification of the relevant peptides was not performed, and the phosphorylation site localization may have some effect on the ionization efficiency of the peptides in the mass spectrometer (thus affecting their measured intensity), the vast differences in measured intensities of these peptides suggest that Tyr185 + Thr188 and Thr183 + Tyr185 are the most abundant populations of doubly phosphorylated peptides. The doubly phosphorylated Thr183 + Thr188 peptide was below the MS detection limit, thus at least several orders of magnitude lower than the other doubly phosphorylated peptides, suggesting its rare occurrence or even absence. Triply phosphorylated molecules, phosphorylated on Thr183 + Tyr185 + Thr188, were identified only among Erk2^R65H^ molecules at a relatively low intensity (Table 1).

As the activity of singly phosphorylated Erk proteins was shown to be extremely low (50), it is almost certain that the dually phosphorylated molecules within the population are responsible for the intrinsic activity of Erk molecules mutated at the αC-helix Arg. The question remains whether not only molecules that are phosphorylated on the TEY motif but also those phosphorylated on Tyr185 + Thr188, which are the majority of the dually phosphorylated molecules in the populations of Erk2^R65S^ and Erk2^R65H^, are catalytically active. Attempts to isolate and separate each type of dually phosphorylated protein and test its catalytic capability was not successful, leaving the question open. In any case, the MS/MS analysis showed that mutating Arg84/65 allowed phosphorylation of any of the three phosphor-acceptors of the activation loop (Table 1).

### Arg84/65 of Erk1/2 is invariant in all MAPKs but is not conserved in other EPKs

**Figure 7:**
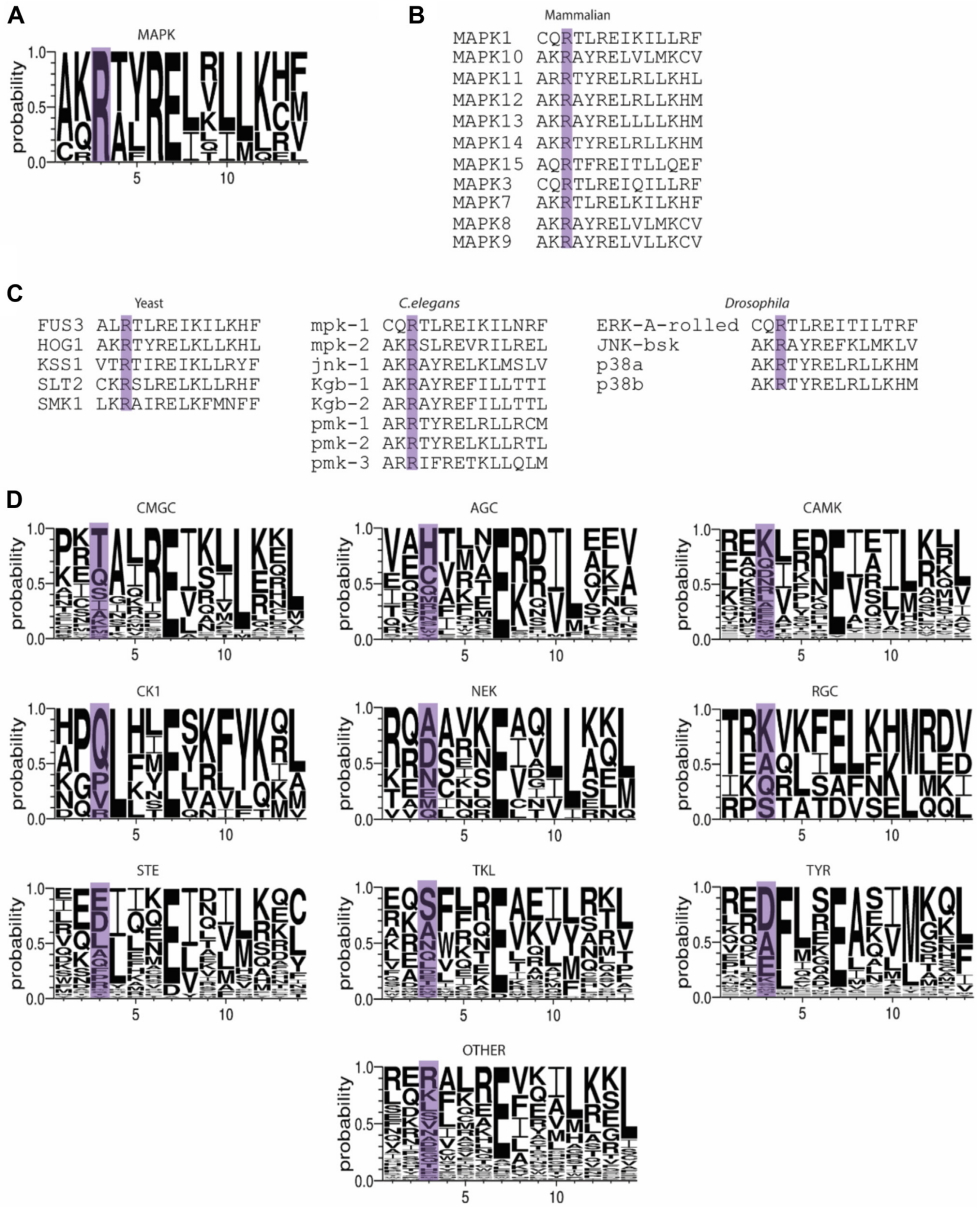
**An Arg residue, equivalent to Arg84/65 of Erk1/2, is invariantly conserved in MAPKs, found in all MAPKs in nature, and is restricted to MAPKs.***A*, a WebLogo depicting the MSA of the α-C helix of all MAPKs. The *y*-axis represents the probability score. The *x*-axis displays the position of amino acid in the MSA. The αC-helix Arg is highlighted in *light purple*. Note that it is present in all MAPKs. *B*, structure-based MSA found in Kincore was used to align mammalian MAPKs showing 100% conservation of the αC-helix Arg (highlighted in *light purple*). *C*, structure-based MSA of MAPKs found in lower eukaryotes also showing conservation of the αC-helix Arg (highlighted in *light purple*). *D*, WebLogos depicting the MSA of the different EPK families. The position equivalent to Arg84/65 of Erk1/2 in each family in highlighted in *light purple*. EPK, eukaryotic protein kinase; MAPK, mitogen-activated protein kinase; MSA, multiple sequence alignment.

The importance of Arg84/65 of Erk1/2 and Arg68 of Mpk1 as suppressors of autophosphorylation raises the questions if this particular position within the αC-helix has a universal role in other EPKs, and whether there is a correlation between the residue at this position and the ability of a kinase to autophosphorylate. In other words, are other EPKs that possess an Arg at this position also incapable of autocatalysis? By applying a structure-based multiple sequence alignment, generated by Modi and Dunbrack (51), it was revealed that an Arg in this position is invariantly conserved in all MAPKs (Fig. 7*A*). Impressively, this Arg appears in all mammalian MAPK subfamilies (p38s, JNKs, and BMK/ERK5), in all isoforms (Fig. 7*B*) and in all MAPKs of lower eukaryotes, including yeast, *Caenorhabditis elegans*, and *Drosophila melanogaster* (Fig. 7*C*). We could not find a single MAPK that does not harbor an Arg at the relevant position. We therefore refer to this arginine as the MAPK-αC-Arg. The conservation of this Arg is further impressive given that there is no conservation of the sequence of the αC-helix amongst EPKs, and the only highly conserved residue is the glutamic acid (position 7 at Fig. 7*D*), which is critical for interacting with the Lys residue of β3 and stabilizing the active conformation.

Unlike MAPKs, other EPK superfamilies possess various residues at the equivalent position (Fig. 7*D*), suggesting that the rule for an Arg at this position is restricted to MAPKs. Even within the CMGC kinases (the group to which MAPKs belong), this Arg is not conserved. For instance, CDKs possess either a threonine or a serine (except for CDK20 that has a glutamine), CLKs have an alanine, GSK3s possess a phenylalanine, and DYRKs have a glutamine. The only kinase among members of the CMGC family that does not belong to the MAPK family and possesses an arginine at this position is Nemo-like kinase (Supplemental File 1). Nemo-like kinase shares many properties with MAPKs and is actually considered as an atypical member of the MAPK group (52).

Similarly, among the 62 members of the AGC group of EPKs, there is no highly preferred amino acid at this position. About 33.9% of them have histidine (Fig. 7*D*). All members of the MRCK, DMPK, and PKC (apart from PRKCI and PRKCZ) families, belonging to the AGC kinases, harbor a cysteine in this position (Fig. 7*D* and Supplemental File 1). Interestingly, four isoforms of the ribosomal S6 kinase, RPS6KA1, 2, 3, 6, which are activated by Erks, are the only AGCs to have an Arg, equivalent to ERKs’ Arg84/Arg65 (Fig. 7*D* and Supplemental File 1).

In CAMK family, which is one of the largest EPK families, containing 92 members, this position is occupied by many different residues with lysine being the most common (Fig. 7*D* and Supplemental File 1). The CAMKs that have an arginine in this position are CAMKK1, PIM1, PLK4, SPEG-2, MKNK1, MKNK2, and MELK. In CK1 kinase family, most members possess a glutamine in this site, and only one, vaccinia-related kinase 3 possess an Arg. In NEK and RGC groups, there seems to be no preference for a specific residue, and none of them have an arginine (Fig. 7*D* and Supplemental File 1).

Interestingly, in the STE family, this position is mostly occupied by either an acidic amino acid (aspartic or glutamic acid) or leucine. For example, in PAK members, there is a leucine in this position, and in STK members, there is aspartic acid or glutamic acid. Three members of the MAP2K family (MAP2K3, 6, and 7, also known as MEK or MAPKK) have arginine in this position. These kinases are known to phosphorylate MAPKs.

Similar to NEK and RGC families, in the TKL group, there is no preference for a specific residue (Fig. 7*D* and Supplemental File 1). The only member of this family that possesses an arginine in this position is interleukin-1 receptor–associated kinase 3.

In TYR kinases, which form the largest EPK family, containing 94 kinases, no arginine is found in this position (Fig. 7*D* and Supplemental File 1). Notably, these kinases are mostly self-regulated by autophosphorylation. In many Tyr kinases, there is either an acidic residue or an alanine, followed by a hydrophobic residue (mostly Phe, but also Leu, Val, or Met).

The 10th group of typical kinases is known as “OTHER” kinases and consists of the remaining 65 kinases that have a remote homology to other kinase families. Several kinases in this group do have an arginine in this position, including CDC7, CHUK, EIF2AK4, IKBKB, PBK, PKMYT1, RNASEL, STK31, TP53RK, and WNK members. It should be noted that some of these molecules are not *bona fide* kinases.

In summary, the bioinformatics analysis showed that MAPK-αC-Arg is almost exclusively restricted to and invariantly conserved within MAPKs, which almost all of them are incapable of spontaneous autophosphorylation. It is very rarely found in other EPKs, but the few kinases in which it is found may share some properties with MAPKs.

### The MAPK-αC-Arg in Hog1/p38 MAPKs is essential for autophosphorylation

**Supplemental Fig. S4:**
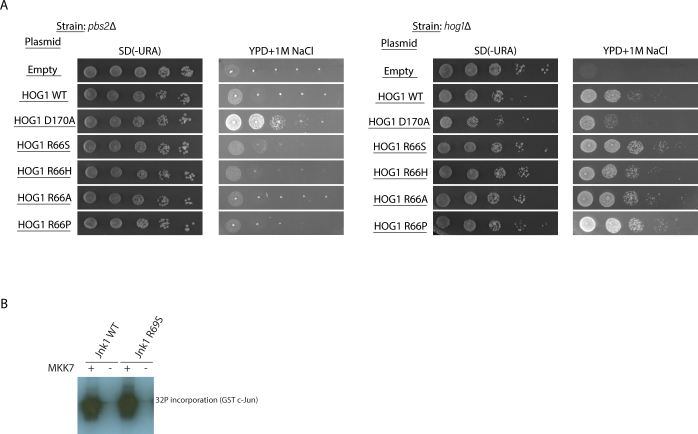
**Mutating Arg66 in Hog1 and Arg69 in JNK1 does not evoke intrinsic activity.** A) *pbs2*Δ (left panel) or *hog1Δ* (right panel) cells harboring the indicated plasmids were plated in five decimal dilutions on plates containing YPD supplemented with 1M NaCl. All strains were also plated on plates containing SD(-URA) medium with no NaCl. B) Recombinant, purified Jnk1^WT^ and Jnk1^R69S^ molecules were tested in an *in vitro* kinase assay using c- Jun as a substrate, with or without pre-treatment with active MKK7. Reaction mixtures were separated on SDS-PAGE and exposed to a X-ray film.

As explained previously, the original mutation in the MAPK-αC-Arg was isolated in a screen in yeast for intrinsically active, MKK1/2-independent, Mpk1 molecules (25). Intriguingly, mutations in the equivalent Arg did not emerge in a similar screen for intrinsically active variants of the MAPK Hog1 (the yeast ortholog of p38) (27, 28), suggesting that in the p38/Hog1 family, it may not be a suppressor of autophosphorylation. As it could be that the screen was not saturated, we tested directly whether mutating Arg66 of Hog1 to Ser, His, Ala, or Pro would render Hog1 intrinsically active. It was found that the mutations did not evoke an intrinsic activity in the resulting mutants (HOG1^R66S^, HOG1^R66H^, HOG1^R66A^, and HOG1^R66P^) (Fig. S4*A*). This suggests that, although highly conserved, the MAPK-αC-Arg of the Hog1/p38 group functions differently than the MAPK-αC-Arg of the Erk group. Attempting to generate intrinsically active variants of JNK1, we also tested mutations in the equivalent Arg in this MAPK (Arg69) and found the resulting mutant, JNK1^R69S^, to behave just like JNK1^WT^, namely manifested catalytic activity only after phosphorylation by MKK7 (Fig. S4*B*). Thus, in JNKs and Hog1/p38s, the Arg equivalent to Arg84/65 of Erk1/2 seems not to function as a major suppressor of autophosphorylation so that modifying it does not derepress autophosphorylation. The fact that HOG1^R66S^, HOG1^R66H^, HOG1^R66A^, HOG1^R66P^, and JNK1^R69S^ function normally, shows that, similar to the observations in Erks, in Hog1/p38s and JNKs too, the MAPK-αC-Arg is not at all required for the MAPKK-dependent activity. Namely, in all kinases tested, it is not essential for stabilizing the active conformations.

**Figure 8:**
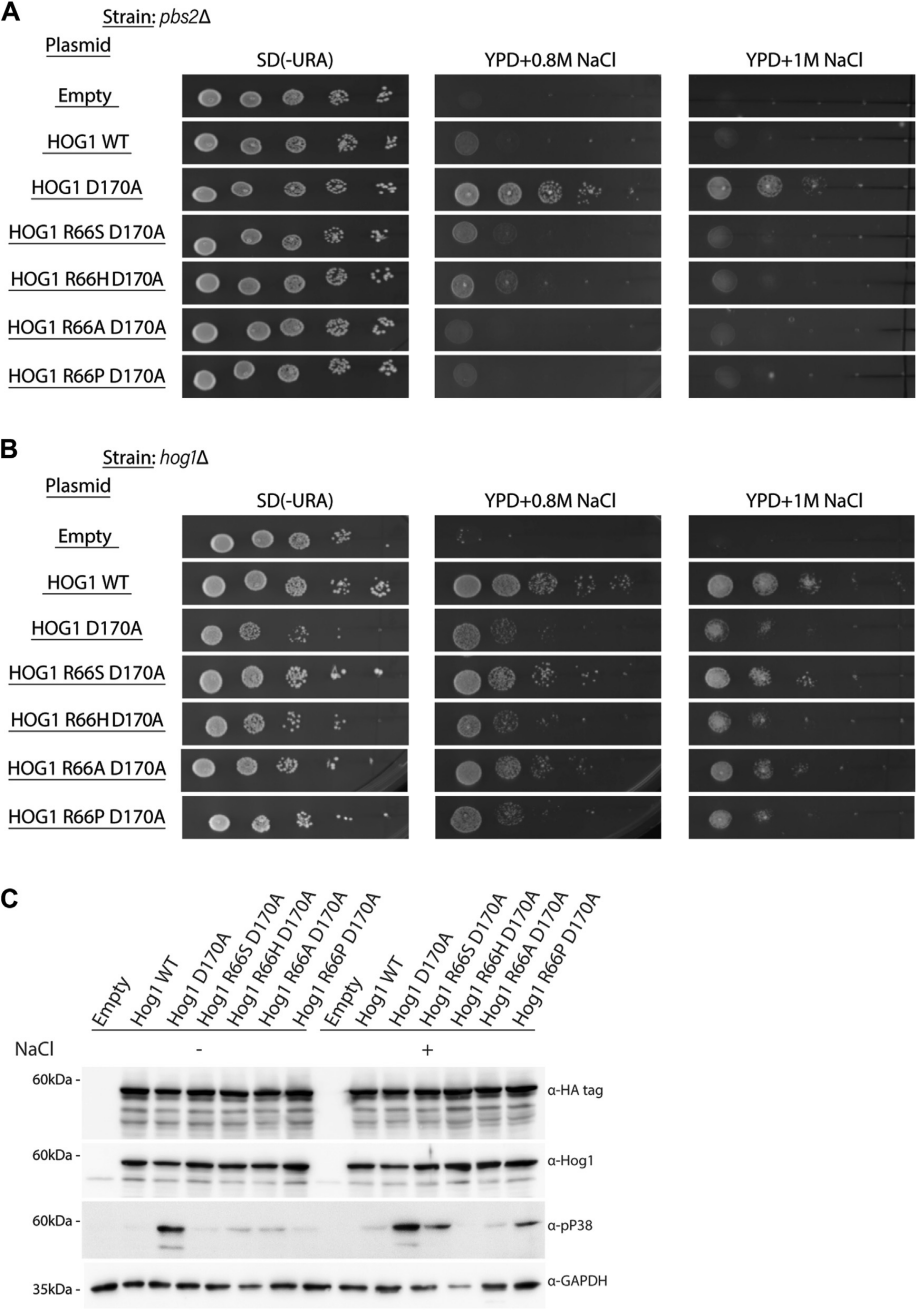
**Mutating Arg66 in the yeast MAPK p38/Hog1 affects specifically intrinsic autophosphorylation but not catalysis.***A*, *pbs2*Δ cells harboring the indicated expression plasmids were plated in five serial dilutions on plates containing YPD supplemented with 0.8 M or 1 M NaCl. All strains were also plated on plates containing SD (−URA) medium with no NaCl. *B*, as in *A* but in *hog1*Δ cells. *C*, protein lysates prepared from *pbs2*Δ cells harboring the indicated plasmids, and exposed or not exposed to 0.77 M NaCl, were subjected to a Western blot analysis and probed using the indicated antibodies. MAPK, mitogen-activated protein kinase; YPD, yeast extract–peptone–dextrose.

**Figure 9:**
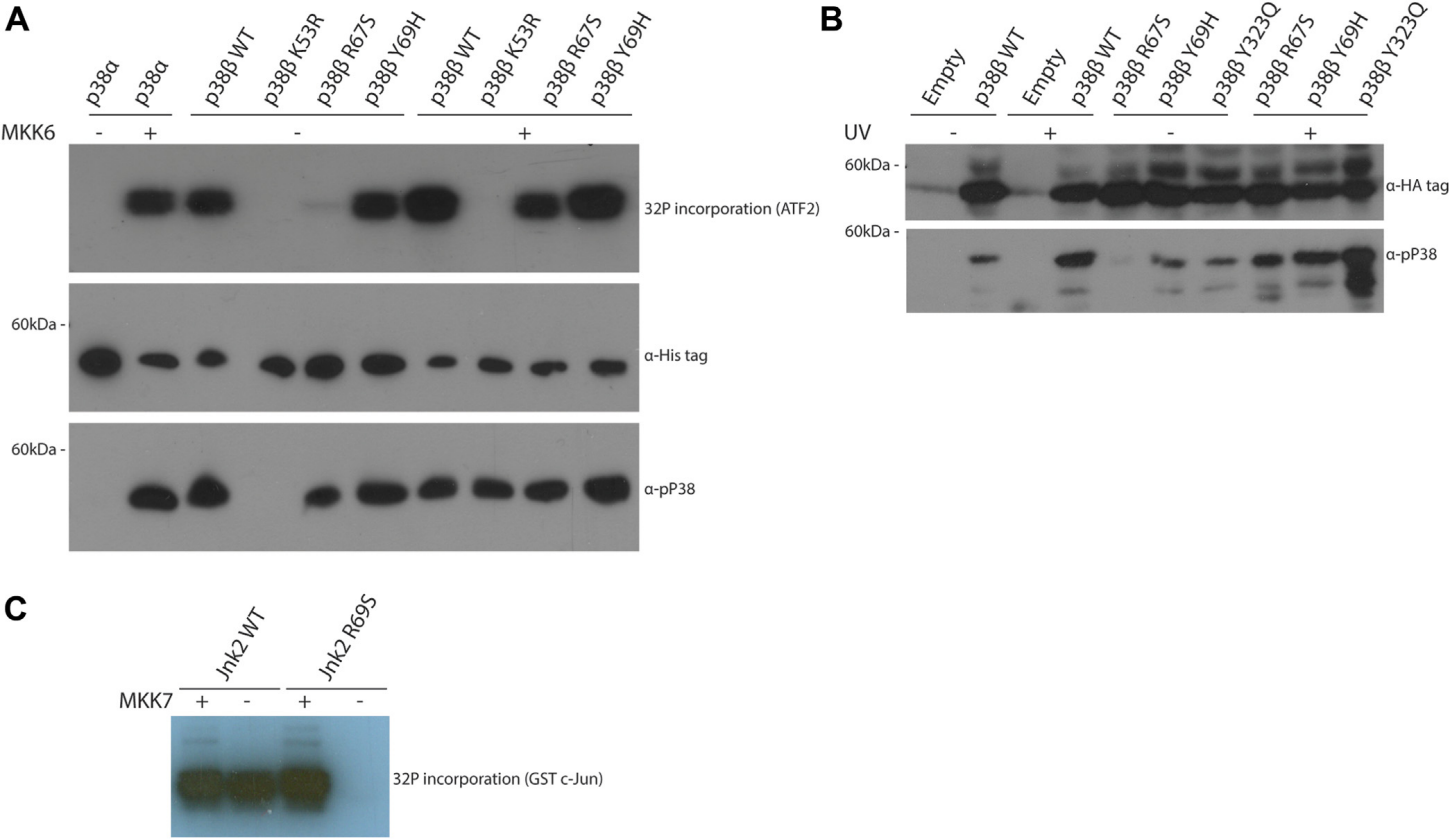
**Arg67 and Arg69 are essential for autophosphorylation of p38β and JNK2α2, respectively.***A*, the indicated, recombinant, purified p38 molecules were tested in an *in vitro* kinase assay. A fixed volume from each reaction was subjected to SDS-PAGE and exposed to an X-ray film to visualize ^32^P incorporation (*first row*). Samples were also subjected to Western blot analysis and probed with antibodies against the polyhistidine, targeting the His tag on the p38β proteins (*second row*), and phosphorylated(TGY) p38 (*third row*). p38β^Y69H^ molecules, mutated in the adjacent residue of Arg67, were used as a control for the specificity of the Arg67 function. *B*, HEK293T cells were transfected with the indicated vectors. About 48 h post-transfection, cells were irradiated, or not, with UV radiation and lyzed 1 h later. Protein lysates were subjected to Western blot analysis using the indicated antibodies. *C*, JNK2α2^WT^ and JNK2α2^R69S^ molecules were tested in an *in vitro* kinase assay using GST-c-Jun as a substrate, with or without pretreatment with active MKK7. Reaction mixtures were separated on SDS-PAGE and exposed to an X-ray film. GST, glutathione-*S*-transferase; HEK293T, human embryonic kidney 293T cell line; JNK, c-Jun N-terminal kinase.

Although not an obvious suppressor of spontaneous autophosphorylation, the MAPK-αC-Arg may still be involved in regulating autophosphorylation of p38s and JNKs. To address this matter, we took advantage of the rare examples of MAPKs that do manifest some degree of spontaneous autophosphorylation. These include p38β (53, 54), the JNK2 splice variant JNK2α2 (55), and the intrinsically active variants of Hog1 (28, 56). The active Hog1 variants are particularly indicative tools for this purpose. These molecules (*e.g.*, Hog1^D170A^ and Hog1^F318S^) acquired intrinsic (MAPKK-independent) catalytic and biological activity. As the Hog1 pathway is essential for proliferation under osmostress, cells knocked out for *HOG1* or for its direct MAPKK *PBS2* would not proliferate on medium supplemented with high concentration of NaCl for example. Hog1 mutants, such as Hog1^D170A^ or Hog1^F318S^, can rescue cells knocked out for *PBS2* (*pbs2Δ* cells), showing that they function independently of MAPKK/Pbs2 activity. The mechanism responsible for the independence of Hog1^D170A^ or Hog1^F318S^ of MAPKK activation is the acquirement of autophosphorylation/autoactivation capabilities (28, 56, 57). Thus, if the MAPK-αC-Arg is critical exclusively for autophosphorylation of these active mutants, then molecules mutated in it will still be activated by MAPKK/Pbs2 but will lose their intrinsic activity. This notion could be tested by monitoring the ability of the mutants to rescue *hog1*Δ and *pbs2*Δ cells of osmostress. If mutating Arg66 in Hog1^D170A^ molecules affects catalysis altogether, the mutants will not be able to rescue either strain. But if mutating Arg66 affects exclusively autophosphorylation, the mutants will still rescue *hog1*Δ but not *pbs2*Δ cells. As is shown in Figure 8*A*, expression of Hog1^D170A^ (but not Hog1^WT^) allows growth of *pbs2Δ* cells in the presence of 0.8 M or even 1 M NaCl, as expected (28, 57). But, Hog1^D170A + R66S^, Hog1^D170A + R66H^, Hog1^D170A + R66A^, and Hog1^D170A + R66P^ could not rescue the osmosensitivity of *pbs2*Δ cells, whereas they did rescue, efficiently, *hog1*Δ cells (Fig. 8, *A* and *B*, lanes 4–7). The loss of their ability to rescue *pbs2*Δ cells is correlated with a reduced level of their phosphorylation on the TGY motif, namely, reduced autophosphorylation activity (Fig. 8*C*). This result suggests that the MAPK-αC-helix of Hog1, Arg66, is specifically essential for autophosphorylation (but not at all for catalysis *per se*). This seems to be the case in the entire Hog1/p38 family because an equivalent mutation in the mammalian p38β R67S had a very similar effect. It abolished p38β′s intrinsic autoactivation capability but maintained its MKK6-dependent activity (Fig. 9*A*). The exclusive role of Arg67 of p38β for autophosphorylation is also observed in cell culture where p38β^WT^, but not p38β^R67S^, manifested spontaneous phosphorylation (Fig. 9*B*). All proteins were phosphorylated in cells exposed to UV radiation, a known stimulator of the p38 pathway (58) (Fig. 9*B*). Thus, similar to the case in the active variants of the yeast Hog1, Arg67 of mammalian p38β is specifically required for intrinsic autophosphorylation and not for the catalytic activity itself.

Finally, we replaced Arg69 in JNK2α2 to Ser and observed that this mutation prevented its autoactivation capabilities, whereas its MKK7-induced activity was maintained (Fig. 9*C*). Thus, similar to the case seen in p38β and in intrinsically active variants of Hog1, the MAPK-αC-helix-Arg of JNK2α2 is important for autophosphorylation but not for catalysis.

In summary, the MAPK-αC-Arg plays a critical role in controlling autocatalysis in all MAPKs, but the exact roles differ between Erks and Hog1/p38s/JNKs, reflecting nuances in the evolution of the control over autophosphorylation within each MAPK family.

### In the crystal structures of JNK2α2 and p38β, both capable of autophosphorylation, MAPK-αC-Arg does not interact with DFG residues

**Figure 10:**
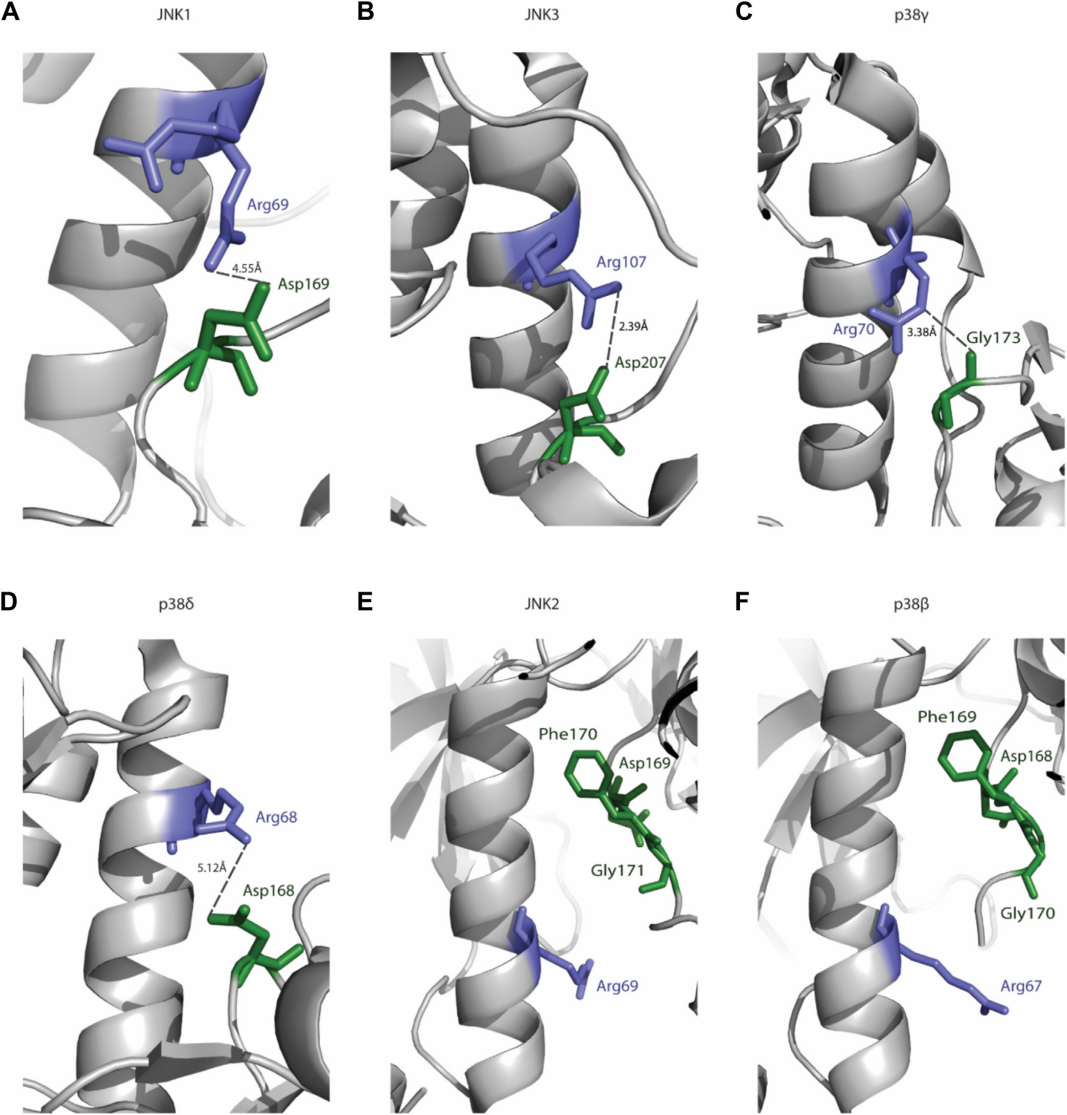
**Analysis of crystal structures of p38s and JNKs reveals an association between interaction of MAPK-αc-helix-Arg with DFG and spontaneous autophosphorylation capability.***A*, amino group of Arg69 (colored in *purple*) of JNK1 (Protein Data Bank [PDB] code: 4L7F) interacts with the carboxyl group of Asp169 (colored in *green*) of the DFG motif. The distance of the charged interaction between the two atoms is indicated. *B*–*D*, as in *A*, but a zoom in into the interaction between the MAPK-αc-Arg and DFG motif of JNK3 (PDB code: 4KKG), p38γ (PDB code: 7CGA), and p38δ (PDB code: 4YNO). *E*, zoom in into the MAPK-αc-Arg (colored in *purple*) of JNK2 (PDB code: 3E7O) showing no association with the DFG motif (colored in *green*), providing a potential analysis for its autophosphorylation activity. *F*, as in *E* but the structural analysis of p38β (PDB code: 3GC9). JNK, c-Jun N-terminal kinase; MAPK, mitogen-activated protein kinase.

As discussed, the MAPK-αC-Arg of ERKs interacts with the DFG motif (Fig. 5). To test whether in other MAPKs it also associates with the DFG, we analyzed available crystal structures of MAPKs. In JNK1 (PDB code: 4L7F, with inhibitor), the arginine has two possible conformations, which indicate that, as in Erk2, it is also a flexible residue. Interestingly, similar to the case observed in Erk2 structures, in one conformation, the MAPK-αC-Arg of JNK1 (Arg69) is rotated toward the Asp of the DFG motif and can form a charged interaction with it (Fig. 10*A*). In the apo-structure of JNK3 (PDB code: 4KKG), the MAPK-αC-Arg (Arg107) can interact with Asp207 of the DFG motif (Fig. 10*B*). In inactive p38γ structure (PDB code: 7CGA), MAPK-αC-Arg, Arg70, interacts with Gly173 of the DFG motif (Fig. 10*C*). Similarly, in p38δ structure (PDB code: 4YNO), Arg68 can make hydrogen bonds with Asp168 of the DFG motif (Fig. 10*D*). Interestingly, MAPK-αC-Arg does not interact with DFG residues in the crystal structures of JNK2α2 and p38β, both capable of autophosphorylation (Fig. 10, *E* and *F*). In the JNK2α2 structure (PDB code: 3E7O) (59), the conserved Arg69 side chain can interact with His66. It is not oriented toward the DFG motif and makes no interaction with it (Fig. 10*E*). Similarly, in the three available p38β costructures with inhibitors, the conserved Arg67 makes only backbone interactions with residues from the αC-helix and does not interact with the DFG motif (Fig. 10*F*). Taking together, the observations in the structures of Erk2^R65S^, Erk2^I84A^, JNK2, and p38β propose that the absence of the interaction between the αC-helix Arg and the DFG is a potential explanation for the spontaneous autophosphorylation activity of these molecules. Nevertheless, the absence of this interaction is not sufficient for promoting autophosphorylation as in the structures of p38α (PDB codes: 5ETC and 5UOJ), which does not manifest basal activity, the conserved Arg67 makes no interaction with the DFG motif.

We thus propose that disrupting the interaction of the MAPK-αC-helix Arg with the DFG motif is an important event, but not always sufficient, for igniting autophosphorylation of MAPKs.

In summary, the experimental results, combined with structural and bioinformatic analysis, point at the position at the αC-helix, occupied by histidine (His87) in cPKA and Arg84/65 in Erk1/2, as a specific regulator, of autophosphorylation, and not of catalysis *per se*, of MAPKs.

The fact that an Arg at this position is MAPK specific distinguishes between MAPKs and the rest of EPKs. Analysis of the position in all other EPKs is required to assess the effect of this site, if any, on the activity of each EPK, particularly on its ability to acquire the “prone-to-autophosphorylate” conformation. It was already shown that in cPKA, conversion of His87 to Ala caused an increase in intrinsic activity, which relies on autophosphorylation (19). This clearly shows that His87 is not critical for catalysis and seems to be involved in autophosphorylation, supporting an idea that the nature of the residue at the position may affect the autophosphorylation properties of many EPKs.

Why did evolution preserve an efficient autophosphorylation potential (even if suppressed) in the Erk1/2 molecules, which are activated by MEK and do not seem to need autophosphorylation for activation? The answer to this riddle is currently unknown, but in the p38 family, the α, β, and γ isoforms were reported to be activated, under specific physiological conditions, *via* the so called “alternative pathway,” a machinery that exploits their hidden autophosphorylation capacity (31, 32, 33). Perhaps Erk molecules too are activated, under yet unknown situations, by desuppressing their autophosphorylation potential. The observation that Erk2 is phosphorylated on Thr188 in patients with cardiac hypertrophy (43, 49) and in cells derived from tumors (Fig. S3), combined with the identification of the R84H mutation in ERK1 of some cancer patients, supports such a notion. Understanding the alternative mode of Erk activation is therefore clinically important for both cancer and cardiac diseases, and inhibitors against it, particularly against Erk1^R84H^, might be new tools for combating these morbidities.

## Experimental procedures

### Cloning and plasmids

Previously reported pET15b or pCEFL vectors carrying either human ERK1 or rat ERK2 (6, 25) were used as bacterial and mammalian expression vectors and for templates for site-directed mutagenesis. Mutants generated in pET15b were N-terminally tagged by a 6xHis tag. ERK1 mutants in pCEFL were also N-terminally tagged with a 6xHis tag, whereas ERK2 mutants in pCEFL were N-terminally tagged with 3xHA tag (YPYDVPDYA). For expression in yeast and for generating mutations in *MPK1* and *HOG1*, the plasmids pAES426-HA-MPK1^WT^ and pAES426-HA-HOG1^WT^ (25) were used. For bacterial and mammalian expression and for mutating p38β, we used pET15bp38β^WT^ and pCEFL-3xHA-p38β^WT^ vectors (53). For bacterial expression, human JNK2α2 complementary DNA (N-terminally tagged with 6xHis) was cloned into the NdeI/HindIII restriction sites of pET28. Site-directed mutagenesis of all genes was performed using the quick-change mutagenesis kit, according to the manufacturer’s instructions, using the PfuUltraII Fusion HS DNA Polymerase (Agilent; catalog no.: 600672). Primers used are listed in Table 2.

**Table 2:** Primers used in site-directed mutagenesis reactions

Description	Primer sequence
ERK1-R84H-F	CCTACTGCCAGCACACGCTCCGGGAG
ERK1-R84H-R	CTCCCGGAGCGTGTGCTGGCAGTAGG
ERK1-R84Y-F	CAGACCTACTGCCAGTACACGCTCCGGGAGATC
ERK1-R84Y-R	GATCTCCCGGAGCGTGTACTGGCAGTAGGTCTG
ERK1-R84A-F	CAGACCTACTGCCAGGCCACGCTCCGGGAGATC
ERK1-R84A-R	GATCTCCCGGAGCGTGGCCTGGCAGTAGGTCTG
ERK1-R84T-F	CAGACCTACTGCCAGACCACGCTCCGGGAGATC
ERK1-R84T-R	GATCTCCCGGAGCGTGGTCTGGCAGTAGGTCTG
ERK1-R84P-F	CAGACCTACTGCCAGCCCACGCTCCGGGAGATC
ERK1-R84P-R	GATCTCCCGGAGCGTGGGCTGGCAGTAGGTCTG
ERK1-T84K-F	CAGACCTACTGCCAGAAAACGCTCCGGGAGATC
ERK1-T84K-R	GATCTCCCGGAGCGTTTTCTGGCAGTAGGTCTG
ERK2-R65H-F	CAGACCTACTGTCAGCATACCCTGAGAGAG
ERK2-R65H-R	CTCTCTCAGGGTATGCTGACAGTAGGTCTG
ERK2-R65A-F	CACCAGACCTACTGTCAGGCAACCCTGAGAGAGATAAAA
ERK2-R64A-R	TTTTATCTCTCTCAGGGTTGCCTGACAGTAGGTCTGGTG
ERK2-R65T-F	CCAGACCTACTGTCAGACAACCCTGAGAGAGATAA
ERK2-R65T-R	TTATCTCTCTCAGGGTTGTCTGACAGTAGGTCTGG
ERK2-R65Y-F	GCACCAGACCTACTGTCAGTATACCCTGAGAGAGATAAAAA
ERK2-R65Y-R	TTTTTATCTCTCTCAGGGTATACTGACAGTAGGTCTGGTGC
ERK2-R65P-F	CCAGACCTACTGTCAGCCAACCCTGAGAGAGATAA
ERK2-R65P-R	TTATCTCTCTCAGGGTTGGCTGACAGTAGGTCTGG
ERK2-R65K-F	CCAGACCTACTGTCAGAAAACCCTGAGAGAGATAA
ERK2-R65K-R	TTATCTCTCTCAGGGTTTTCTGACAGTAGGTCTGG
MPK1-R68S-F	AAGACCTTACTATGTAAAAGCTCCCTACGTGAGCTAAAG
MPK1-R68S-R	CTTTAGCTCACGTAGGGAGCTTTTACATAGTAAGGTCTT
MPK1-R68H-F	AAGACCTTACTATGTAAACACTCCCTACGTGAGCTAAAG
MPK1-R68H-R	CTTTAGCTCACGTAGGGAGTGTTTACATAGTAAGGTCTT
MPK1-R68A-F	AAGACCTTACTATGTAAAGCATCCCTACGTGAGCTAAAG
MPK1-R68A-R	CTTTAGCTCACGTAGGGATGCTTTACATAGTAAGGTCTT
MPK1-R68T-F	AAGACCTTACTATGTAAAGAATCCCTACGTGAGCTAAAG
MPK1-R68T-R	CTTTAGCTCACGTAGGGATGTTTTACATAGTAAGGTCTT
MPK1-R68Y-F	AAGACCTTACTATGTAAATACTCCCTACGTGAGCTAAAG
MPK1-R68Y-R	CTTTAGCTCACGTAGGGAGTATTTACATAGTAAGGTCTT
MPK1-R68P-F	AAGACCTTACTATGTAAACCCTCCCTACGTGAGCTAAAG
MPK1-R68P-R	CTTTAGCTCACGTAGGGAGGGTTTACATAGTAAGGTCTT
MPK1-R68K-F	AAGACCTTACTATGTAAAAAGTCCCTACGTGAGCTAAAG
MPK1-R68K-R	CTTTAGCTCACGTAGGGACTTTTTACATAGTAAGGTCTT
MPK1-R68E-F	AAGACCTTACTATGTAAAGAATCCCTACGTGAGCTAAAG
MPK1-R68E-R	CTTTAGCTCACGTAGGGATTCTTTACATAGTAAGGTCTT
HOG1-R66S-F	CCACTGCAGTGCTGGCCAAAAGCACATATCGTGAAC
HOG1-R66S-R	GTTCACGATATGTGCTTTTGGCCAGCACTGCAGTGG
HOG1-R66H-F	CCACTGCAGTGCTGGCCAAACATACATATCGTGAAC
HOG1-R66H-R	GTTCACGATATGTATGTTTGGCCAGCACTGCAGTGG
HOG1-R66A-F	CCACTGCAGTGCTGGCCAAAGCGACATATCGTGAAC
HOG1-R66A-R	GTTCACGATATGTCGCTTTGGCCAGCACTGCAGTGG
HOG1-R66P-F	CCACTGCAGTGCTGGCCAAACCGACATATCGTGAAC
HOG1-R66P-R	GTTCACGATATGTCGGTTTGGCCAGCACTGCAGTGG
JNK2-R69S-F	CCAAACTCATGCAAAGAGCGCTTATCGTGAACTTGTCC
JNK2-R69S-R	GGACAAGYYCACGATAAGCGCTCTTTGCATGAGTTTGG
P38β-R67S-F	CGCTGATCCACGCGCGCAGCACGTACCGGGG
P38β-R67S-R	CCCGGTACGTGCGCGCGTGGATCAGCG
P38β-Y68H-F	CACGCGCGCAGAACGCACCGGGAGCTGCGG
P38β-Y68H-R	CCGCAGCTCCCGGTGCGTTCTGCGCGCGTG
P38β-Y323Q-F	CGCTCTCATCTTGTGGCTCGGCCTCTGGCTCATCCTCGGGG
P38β-Y323Q-R	CCCCGAGGATGAGCCAGAGGCCGAGCCACAAGATGAGAGCG

### Protein expression and purification

All proteins were expressed and purified as previously described (25). Briefly, expression plasmids were introduced into *E. coli* Rosetta strain cells (Novagene), which were grown at 37 ^o^C in LB medium to logarithmic phase. Expression was induced at 30 °C with 0.2 mM IPTG, and cultures were further incubated for 5 h. Cells were harvested by centrifugation at 3200*g* for 10 min and flash frozen at −80 °C. Protein purification was performed using Ni–NTA beads (Hadar Biotech). Proteins were dialyzed, and their concentrations were determined based on their extinction coefficient at 280 nm. Finally, they were flash frozen in liquid nitrogen and stored at −80 °C.

### *In vitro* kinase assay

*In vitro* kinase assay of all proteins was done as described previously (6). In brief, Erk1/2 proteins were incubated with [γ-^32^p] ATP and the substrate myelin basic protein, with or without pretreatment with active MEK1. p38β activity was checked toward the substrate glutathione-*S*-transferase-ATF2, with or without pretreatment with active MKK6. JNK2 activity was measured toward glutathione-*S*-transferase-c-Jun, with or without preactivation with active MKK7. The activity was quantified by monitoring ^32^P incorporation into the substrates using a scintillation counter running a ^32^P Cherenkov program and was also visualized, qualitatively by running a fraction of the reaction mixture on SDS-PAGE. Other fractions of reaction mixtures were tested by Western blot.

### Mammalian cell culture transfection and foci formation assay

HEK293T, NIH3T3, PC3, and PANC-1 cells were cultured in Dulbecco's modified Eagle's medium (Sigma–Aldrich), whereas Karpas and Jurkat cells were grown in RPMI1640 (Gibco) medium. All media were supplemented with 10% fetal bovine serum, sodium pyruvate, penicillin, and streptomycin (Biological Industries). In the case of PC3, nonessential amino acids were included in the media. Cells were grown at 37 °C and 5% CO_2_. HEK293 cells were transfected using the calcium phosphate method (60). About 48 h post transfections, cells were serum starved (incubated in medium with no serum) for 16 h. Then, cells were either exposed to a fresh medium or to a medium containing 50 ng/ml EGF (PeproTech; catalog no.: AF-100-15) for 10 min. To prepare cell lysates, medium was removed, cells were washed twice with cold PBS, and then scrapped into tubes with 100 μl of Laemmli sample buffer (1.075 M glycerol, 2% SDS, 0.05 M Tris [pH 6.5], 0.1 M DTT, and bromophenol blue dye) using a rubber policeman followed by 10 min of boiling at 95 °C. Protein concentration was measured using the protein quantification assay (Macherey–Nagel; catalog no.: 740967.50).

### Foci formation assay

NIH3T3 cells were transfected using TurboFect transfection reagent (Thermo Scientific; catalog no.: R0533) according to the manufacturer's instructions. About 48 h post-transfection, cells were selected for the presence of the plasmids by incubation with 400 μg/ml G-418 (Sigma–Aldrich; catalog no.: A1720). Four weeks after transfection, cells were washed with PBS, fixed with 100% MeOH for 20 min, and stained with 4% crystal violet dye for 5 min.

### Monitoring p38β activity in HEK293 cells

About 48 h post-transfection, cells were irradiated by UV (120 J/m^2^) and lysed 1 h later. Samples were separated by SDS-PAGE and subjected to Western blot analysis with α-HA and α-p-p38 antibodies.

### Luciferase assay

About 7.5 × 10^5^ HEK293T cells were cotransfected with an ERK expression vector, Renilla luciferase, and either 6× AP-1-luc construct or pY2-luc construct (6). The assay was performed 24 h post-transfection. Cells were lysed according to the manufacturer’s protocol (Promega; dual luciferase assay; catalog no.: E1910), and luciferase was measured with a plate luminometer (Plate Reader-BioTek Synergy H1). The firefly luciferase activity was normalized to the Renilla luciferase activity.

### Yeast strains, media, and cell lysate preparation

The *Saccharomyces cerevisiae* strains used in this study were *mkk1Δ/mkk2Δ* (BY4741; *Mat a; his3Δ1; leu2Δ0; met15Δ0; ura3Δ0, YPL140c::kanMX4*, *mkk1::LEU2;* (25)), *mpk1Δ* (BY4741; *Mat a; his3Δ1; leu2Δ0; met15Δ0; ura3Δ0; YHR030c::kanMX4*; obtained from EUROSCARF), JBY13 (*MATa, ura3-52, lys2-801*^*amber*^*, ade2-101*^*ochre*^*, trp1-Δ63, his3-Δ200, leu2-Δ1, hog1Δ::TRP1*), and MAY1 (*MATa, ura3-52,lys2–801*^*amber*^*,ade2–101*^*ochre*^*, leu2-Δ1,his3-Δ200,pbs2-Δ2::LEU2*). JBY13 and MAY1 strains were obtained from M.C. Gustin, Rice University. Yeast cultures were maintained on YPD (1% yeast extract, 2% Bacto Peptone, and 2% glucose, plus, where indicated, 14–15 mM caffeine or 0.8–1.0 M NaCl) or on the synthetic medium YNB(-URA) (0.17% yeast nitrogen base without amino acids and NH_4_(SO_4_)_2_, 0.5% ammonium sulfate, 2% glucose, and 40 mg/l adenine, histidine, tryptophan, lysine, leucine, and methionine).

### Yeast cell plasmid transformation and drop assay

Plasmids were transformed into the relevant *S. cerevisiae* strains by the lithium acetate method (61).

Drop assay of yeast cells on medium supplemented with caffeine or NaCl was performed as previously described (62).

### Protein lysate preparation of yeast cultures

To induce osmotic shock in liquid media, cultures were grown to logarithmic phase (absorbance of 0.5–1.0 at 600 nm) at 30 °C. Culture was split in half, where half of the culture was supplemented with 0.77 M NaCl and the other half received double-distilled water. Cells were collected 10 min later (3500*g*, 5 min) and incubated with 0.1 N NaOH for 5 min, followed by centrifugation at 17,000*g* for 3 min. About 100 μl Laemmli sample buffer was added on the cell pellet, and the samples were then boiled at 97 °C for 5 min. Protein concentration was measured using the protein quantification assay (Macherey–Nagel; catalog no.: 740967.50). Finally, 10 μg of protein were loaded onto 12% SDS-PAGE and subjected for Western blot analysis.

### Western blot

Unless stated otherwise, 30 μg of the different lysates were separated on 12% SDS-PAGE, transferred to polyvinylidene difluoride membranes (Bio-Rad; catalog no.: 1620174) and blocked in blocking buffer, composed of Tris-buffered saline (TBS)  (10 mM Tris–HCl [pH 7.5] and 150 mM NaCl) with 0.1% Tween-20 (TBS-T) supplemented with 3% dry milk. Blots were then probed with primary antibodies diluted 1:1000 in blocking buffer overnight at 4 °C. Following three washes with TBS-T, the membranes were incubated with secondary antibody diluted 1:10,000 in blocking buffer for 1.5 h at room temperature and then washed three times with TBS-T. Membranes were incubated with ECL mix (Thermo Fisher Scientific) for 2 min, and reactive bands were visualized using Fusion Pulse (Vilber Lourmat). Antibodies used in this study are listed later (Table 3).

**Table 3:** Antibody list used in this study

Antibody	Manufacturer
α-ERK	Cell Signaling Technology; catalog no.: 4695
α-p(TEY) ERK	Cell Signaling Technology; catalog no.: 4370
α-p(T207/188) ERK	Kinexus; catalog no.: pk865
α-His	Sigma; catalog no.: H1029
α-HA	Roche; catalog no.: 11867423001
α-Mpk1	Santa Cruz Biotechnology; catalog no.: SC6803
α-Hog1	Santa Cruz Biotechnology; catalog no.: SC9079
α-p-p38	Cell Signaling Technology; catalog no.: 9211
α-GAPDH	Thermo Fisher Scientific; catalog no.: MA515738

### Targeted proteomic analysis

To validate and confirm identification of specific phosphopeptides within the Erk molecules in an unambiguous manner, eight synthetic peptides that correspond to residues 171 to 189 of Erk1 (^171^-VADPDHDHTGFLTEYVATR-^189^) were synthesized with heavy-isotope labeled amino acids (heavy labeled Arg∗ = Arg U-13C6, U-15N4) (JPT Innovative Peptide Solutions). The synthetic peptides included a nonphosphorylated, monophosphorylated (on either Thr183, Tyr185, or Thr188), dually phosphorylated (Thr183 + Tyr185; Thr183 + Thr188; Tyr185 + Thr188), and a triply phosphorylated (on all three residues) peptide. Synthetic peptides were included in the analysis to unequivocally identify each peptide.

### Sample preparation

Protein samples were subjected to in-solution tryptic digestion using the suspension trapping (S-Trap) method as previously described (63). Briefly, 6 μg of total protein was supplemented with a 10% SDS stock solution to a final 5% SDS concentration. The protein was reduced with 5 mM DTT and alkylated with 10 mM iodoacetamide in the dark. Each sample was loaded onto S-Trap micro-columns (ProtiFi) according to the manufacturer’s instructions. After loading, samples were washed with 90:10% methanol/50 mM ammonium bicarbonate. Samples were then digested with trypsin (1:50 trypsin/protein) for 1.5 h at 47 °C. The digested peptides were eluted using 50 mM ammonium bicarbonate. Trypsin was added to this fraction and incubated overnight at 37 °C. Two more elutions were made using 0.2% formic acid and 0.2% formic acid in 50% acetonitrile. The three elutions were pooled together, desalted using Oasis-HLB μElution format (Waters), and vacuum-centrifuged to dryness. Samples were kept at −80 °C until further analysis.

Stable isotope labeled synthetic peptides (SpikeTides L) were purchased from JPT Peptide Technologies GmbH. Dry peptides were dissolved in 97:3% H_2_O/acetonitrile + 0.1% formic acid.

### Liquid chromatography

ULC/MS grade solvents were used for all chromatographic steps. Dry digested samples were dissolved in 97:3% H_2_O/acetonitrile + 0.1% formic acid. Samples were spiked with equal amounts of isotopically labeled synthetic peptides (after optimization), loaded, and then analyzed using split-less nano-Ultra Performance Liquid Chromatography (10 kpsi nanoAcquity; Waters). The mobile phase was (A) H_2_O + 0.1% formic acid and (B) acetonitrile + 0.1% formic acid. The peptides were loaded and separated using a T3 HSS nanocolumn (75 μm internal diameter, 250 mm length, 1.8 μm particle size; Waters). Loading and elution from the column into the mass spectrometer was done using the following gradient: 3% B for 10 min at 500 μl/min while loop offline (loading), followed by separation at 350 μl/min going from 3% to 35% B in 44 min, 35% to 90% B in 5 min, maintaining at 90% B for 5 min, and then back to initial conditions.

### Mass spectrometry

The nano-Ultra Performance Liquid Chromatography was coupled online through a nanoESI emitter (10 μm tip; New Objective) to a quadrupole orbitrap mass spectrometer (Orbitrap Exploris 480; Thermo Scientific) using a FlexIon nanospray apparatus (Proxeon).

Data were acquired in PRM mode, while monitoring all Erk2 peptides of interest (heavy and light VADPDHDHTGFLTEYVATR with 0, 1, 2, or 3 phosphorylations, as well as light mutant peptides with or without phosphorylations and nonmodified Erk2 peptides GQVFDVGPR, FDMELDDLPK, and ICDFGLAR). Identification of the additional Erk2 peptides to whom no synthetic standards were used was done during method optimization, using a data-dependent acquisition analysis of the samples, while searching the data using the Byonic search engine (64) against the rat Erk2 protein sequence (WT and mutants) and common laboratory protein contaminants.

For the PRM analysis, MS1 resolution was set to 120,000 (at 200 *m/z*), mass range of 375 to 1500 *m/z*, standard automatic gain control target, and maximum injection time was set to 100 ms. MS2 resolution was set to 15,000, quadrupole isolation 2 *m/z*, higher-energy collision dissociation energy 30%, standard automatic gain control target, and maximum injection time mode set to auto.

### Data processing and analysis

Raw data from the PRM experiment were processed with the Skyline software, version 22.2.0.255, which was developed by Michael MacCoss' lab (65). Peak boundaries and relevant transitions were manually refined, and peak intensities were calculated. Peptide intensities were normalized to Erk2 total abundance, as calculated by the sum of the three unmodified Erk2 peptides, which were included in the PRM experiment.

## Data availability

Raw MS/MS data are available at the website of the Institute of Life Science, the Hebrew University of Jerusalem.

## Supporting information

This article contains supporting information.

## Conflict of interest

The authors declare that they have no conflicts of interest with the contents of this article.
