# Supplementary material for: A conserved arginine within the αC-helix of Erk1/2 is a latch of autoactivation and of oncogenic capabilities
Source: J Biol Chem. 2023 Jul 18;299(9):105072. doi: 10.1016/j.jbc.2023.105072 (PMC10458722; doi:10.1016/j.jbc.2023.105072)
Supplement: Supplemental File 1 [file mmc1.docx]

**MSA for all kinase families**

**CMGC family (without MAPK)**


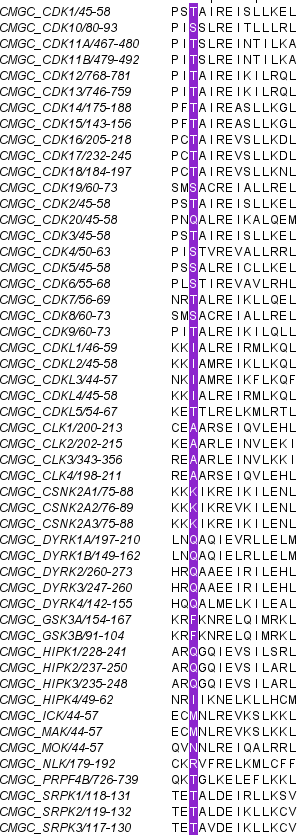


**AGC family**

**
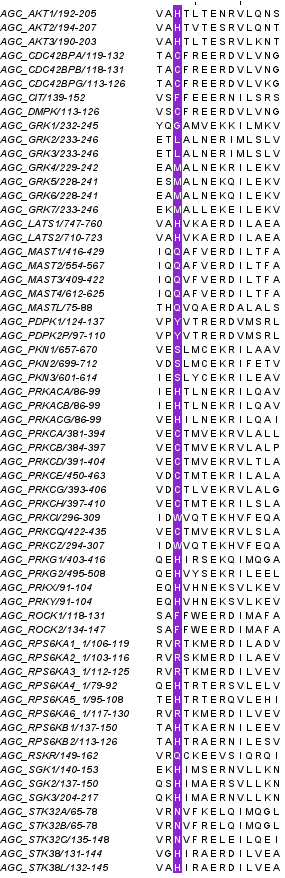
**

**
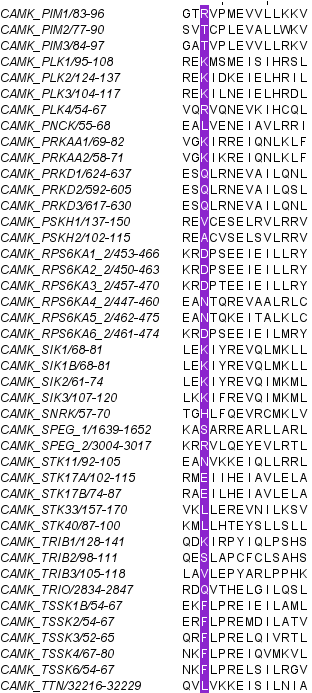

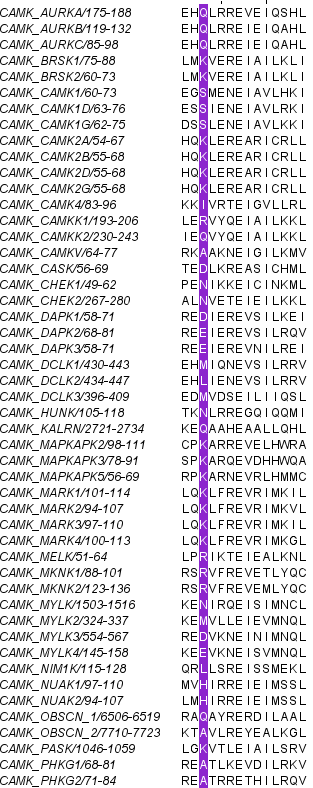
CAMK family**

**CK1 family:**

**
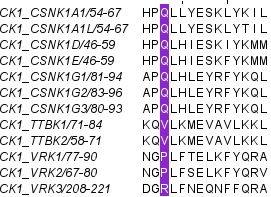
**

**NEK family:**

**
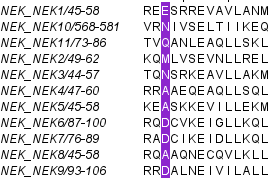
**

**RGC family:**

**
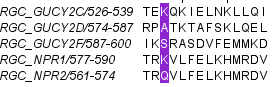
**

**STE family:**

**
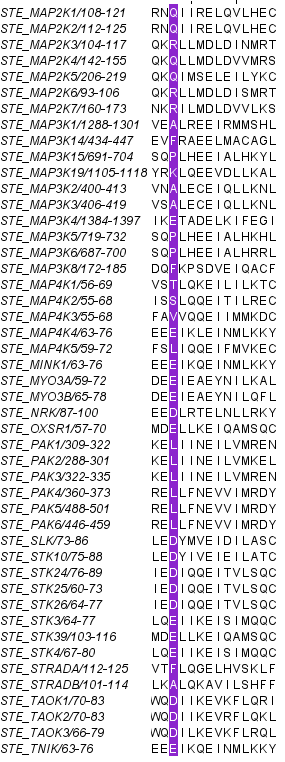
**

**TKL family:**

**
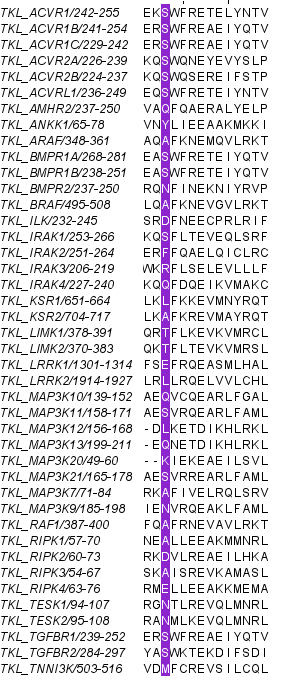
**

**
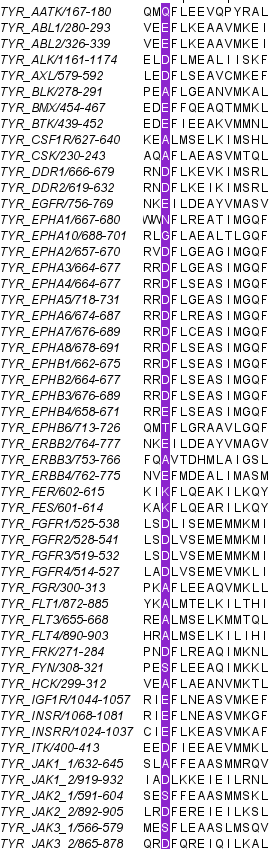
TYR family:**

**
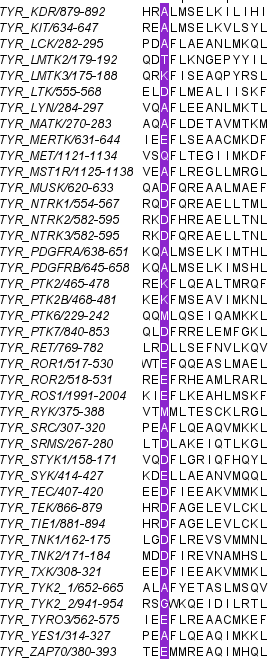
**

**"Other" family:**

**
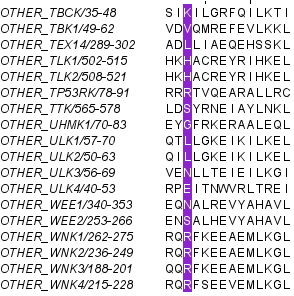

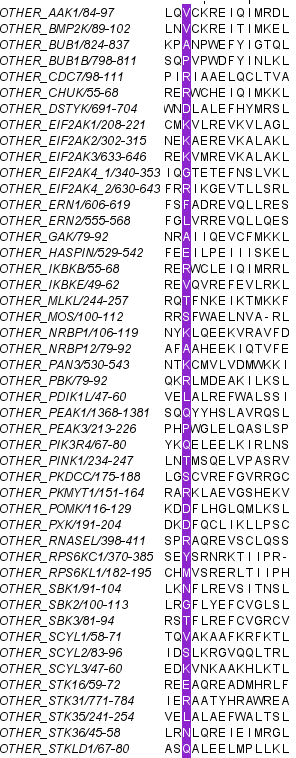
**
